# Resistant Peanut Genotype Reprograms Rhizosphere Metabolism to Enhance Bacterial Wilt Suppression

**DOI:** 10.1002/advs.75910

**Published:** 2026-06-01

**Authors:** Rui Ren, Xiaogang Li, Hao Li, Zenghui Cao, Mengtian Hou, Chunlei Zhao, Nan Lou, Sasa Hu, Yanzhe Li, Qian Ma, Yaoyao Li, Yi Fan, Kunkun Zhao, Kai Zhao, Ding Qiu, Fangping Gong, Zhongfeng Li, Haitao Liu, Xingli Ma, Xingxiang Wang, Dongmei Yin

**Affiliations:** ^1^ College of Agronomy Center For Crop Genome Engineering Henan Agricultural University Zhengzhou China; ^2^ State Key Laboratory of Tree Genetics and Breeding, College of Ecology and Environment Nanjing Forestry University Nanjing China; ^3^ College of Resources and Environment Henan Agricultural University Zhengzhou China; ^4^ State Key Laboratory of Soil and Sustainable Agriculture, Institute of Soil Science Chinese Academy of Sciences Nanjing China

**Keywords:** bacterial wilt, beneficial bacteria, keystone metabolites, peanut, prebiotics, systemic acquired resistance, yield

## Abstract

Bacterial wilt caused by *Ralstonia solanacearum* compromises the yield and quality of peanut (*Arachis hypogaea* L.). While rhizosphere microbiome‐assisted defense is known, how resistant plant genotypes orchestrate this process remains unclear. Here, we integrate multi‐omics analyses of resistant and susceptible peanut genotypes to uncover a genotype‐specific defense mechanism. The resistant genotype selectively recruits beneficial bacteria (e.g., *Kosakonia* and *Frankia*), which coincides with activated salicylic acid (SA)‐dependent systemic acquired resistance (SAR). Crucially, we identify keystone rhizosphere metabolites (including betaine, arginine, and SA) that are positively correlated with both beneficial microbiome assembly and SAR gene expression, establishing a self‐reinforcing defense loop. Leveraging these insights, we develop a prebiotic formulation that enhances beneficial microbial recruitment and stimulates SAR. Field trials demonstrate that the prebiotics reduce bacterial wilt incidence from 84.2% to 5.0% and increase yield by 12.9%–20.3%. Collectively, our study reveals a synergistic microbiome‐immune co‐regulation mechanism in peanut and delivers a translatable solution for sustainable disease management.

## Graphical abstract

The resistant peanut genotype selectively recruits beneficial bacteria, which coincides with the activation of salicylic acid (SA)‐dependent systemic acquired resistance (SAR) against *Ralstonia solanacearum*. Keystone rhizosphere metabolites are positively correlated with both beneficial microbiome assembly and SAR gene expression. Leveraging these insights, the metabolite‐prebiotic formulation significantly enhances peanut resistance to BW and yields.

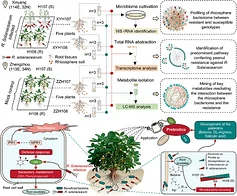

## 1 Introduction

Peanut (*Arachis hypogaea* L.) is a globally important oil and food crop [1, 2]. However, shifts in farming systems, widespread adoption of high‐yield cultivars, and thereby increasingly frequent extreme weather events have collectively intensified soil‐borne diseases and exacerbated the challenges associated with peanut monocropping [3]. Among these diseases, bacterial wilt (BW), caused by *Ralstonia solanacearum*, is particularly destructive [4]. This pathogen induces rapid wilting and plant death through xylem vessel occlusion [5]. *R. solanacearum* infection also acidifies the rhizosphere soil by releasing organic acids, which accelerate root cell wall degradation and disrupts rhizosphere microbial homeostasis [6]. Under conducive environmental conditions, BW can result in severe declines in peanut seed quality and serious yield losses of 50–100% [4, 7, 8].

Peanut resistance to BW primarily involves restricting pathogen proliferation, delaying symptom development, and reducing plant mortality [9, 10]. Several resistance‐associated genes and major quantitative trait loci (QTL) have been identified in a few peanut cultivars, including Yueyou 92 (*qBW‐1*, *qBW‐2*; *AhRLK1*, *AhRRS5*), Yuanza 9102 (*qBWB02.1*, *qBWA12*), and H108 (*AhDef2.2*, *AhNHL24*) [9, 10, 11, 12, 13, 14, 15, 16]. The *R. solanacearum* infection induces a series of rapid responses, including a burst of reactive oxygen species (ROS), accumulation of phytohormones, and reinforcement of the cell wall [17, 18, 19]. Long‐distance signaling of phytohormones leads to activation of systemic acquired resistance (SAR) of plants against a broad range of pathogens [20]. Salicylic acid (SA), abscisic acid, and jasmonic acid signaling, and lignin‐associated cell wall reinforcement have all been implicated in peanut BW resistance, indicating crucial roles of SAR [8, 11, 21, 22, 23, 24]. Nevertheless, broad‐spectrum resistance genes are still limited, and the mechanistic basis of peanut resistance to BW remains incompletely understood.

Plant defense involves both intrinsic immunity and rhizosphere‐mediated protection. Interactions between the plant immune system and rhizosphere microbes enhance the capacity of plants to cope with soil‐borne diseases [25, 26]. The ‘cry for help’ hypothesis proposes that plants under pathogen attack recruit beneficial microbes through root exudates [27, 28]. The higher diversity of the rhizosphere microbial community, the greater the difficulty in the infection of soil‐borne pathogens [29, 30]. Beneficial microorganisms, such as *Pseudomonas spp*., *Chitinophaga spp*., *Chryseobacterium spp*., *Flavobacterium spp*., *Microbacterium spp*., *Sphingomonas spp*., and *Stenotrophomonas spp*., are enriched in the rhizosphere and rescue plants from diverse pathogen attacks [31]. Additionally, those beneficial taxa suppress pathogens through competition with pathogens for space and resources via producing antimicrobial metabolites such as antibiotics, lipopeptides, and phenazines [32, 33]. Certain microbes, such as the *Pseudomonas fluorescens*, trigger SAR against multiple pathogens by promoting the transcription of defense‐related genes [34, 35]. However, the coordinated crosstalk between internal immune pathways and external microbiome modulation in resistant genotypes remains not fully understood.

Rhizosphere metabolites serve as key chemical signals in the ‘cry for help’ process, with specific compounds like coumarins, flavonoids, and benzoxazinoids having been shown to shape protective microbiomes in model plants [36, 37, 38, 39, 40, 41, 42, 43, 44, 45]. Mining the key rhizosphere metabolites is crucial for understanding and harnessing rhizosphere microorganisms‐enhanced resistance against BW in peanuts. Rhizosphere metabolism, microbial colonization, and their dynamic interactions co‐shape the rhizosphere properties of plants under environmental stress conditions [36]. During the interactions, plant roots and microorganisms release diverse rhizosphere metabolites, such as organic acids, alkaloids, carbohydrates, phenols, amino acids, enzymes, antibiotics, flavonoids, phytohormones, and other secondary metabolites [37, 38, 39]. Those rhizosphere metabolites directly affect nutrient cycling, microbiome assembly, and plant healthy growth [36, 40, 41]. Keystone (highly connected and functionally important) rhizosphere metabolites, such as coumarins, flavonoids, indolic compounds, and terpenes, exhibit significant correlation with rhizosphere microorganism growth and root architecture of plants exposed to various biotic and abiotic stresses [42, 43, 44, 45]. Recent advances highlight the potential of metabolite‐based interventions. For instance, taxifolin, a flavonoid compound exudated from onion roots, protects tomato plants against soil‐borne *Verticillium* wilt disease via recruiting of the plant‐beneficial bacteria *Bacillus sp*. in the intercropping system [46]. Development of rhizosphere metabolite‐based prebiotics is a promising approach for controlling soil‐borne diseases. Accordingly, prebiotics/synbiotics have been successfully harnessed for efficient management of BW and aboveground insects [47, 48]. However, the metabolic signatures governing genotype‐specific microbiome assembly remain unclear, and translating these insights into effective field applications presents significant challenges.

**FIGURE 1:**
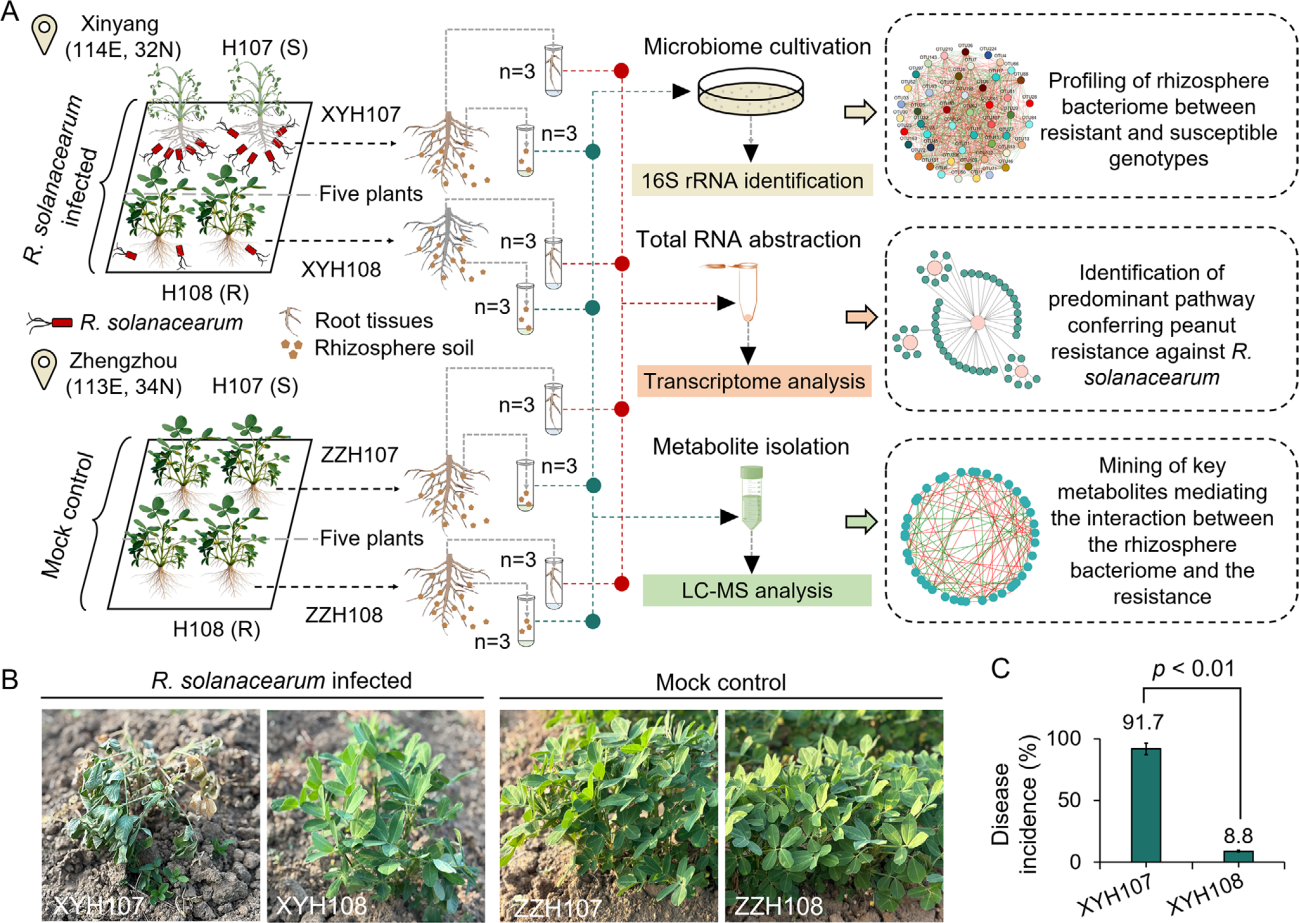
Investigation of factors influencing the occurrence of BW. (A) Multi‐omics workflow for dissecting peanut resistance to BW. E and N denote east longitude and north latitude, respectively. (B) Phenotypes of susceptible (H107) and resistant (H108) genotypes in pathogen‐infested and disease‐free fields. (C) Disease incidences of H107 and H108 plants under *R. solanacearum* infection. Data represent mean ± standard deviation (SD) of three biological replicates; statistical significance was determined by two‐sided Student's *t*‐test (*p* < 0.05).

We hypothesized that the resistant genotype selectively enriches beneficial bacteria through reprogramming rhizosphere metabolites, reinforcing peanut resistance against *R. solanacearum*. To test this hypothesis, we employed a multi‐omics approach comparing the BW‐resistant genotype Nongdahua 108 (H108) and the susceptible genotype Nongdahua 107 (H107) under *R. solanacearum* challenge [15]. We analyzed root transcriptomes, rhizosphere bacteriomes (16S rRNA‐amplicon sequencing), and metabolomes to delineate the interactions among host resistance, microbial recruitment, and metabolic reprogramming (Figure 1A). It is expected to explore and harness key factors enhancing the capability of peanuts to cope with *R. solanacearum* infection.

## 2 Results

### 2.1 Profiling of Rhizosphere Bacteriome Associated with the BW Resistance

To profile the rhizosphere bacteriome associated with the BW resistance, we cultivated resistant (H108) and susceptible (H107) peanut genotypes under field conditions. Peanut plants were grown at a disease‐prevalent site in Xinyang, naturally infested with *R. solanacearum*, and were additionally inoculated with the pathogen *R. solanacearum* (designated XYH107 and XYH108). Meanwhile, H107 and H108 were also grown in a pathogen‐free field in Zhengzhou as mock controls (ZZH107 and ZZH108). Clear differences in disease response were evident by 15 days post‐inoculation (dpi): XYH107 plants displayed severe wilting symptoms, whereas XYH108 and both control groups remained healthy (Figure 1B). By 70 dpi, disease incidence reached 91.7% in XYH107 but only 8.8% in XYH108, confirming strong resistance in H108 and high susceptibility of H107 under pathogen pressure (n = 3 biologically independent replicates; Student's *t*‐test, *p* < 0.05) (Figure 1C).

**FIGURE 2:**
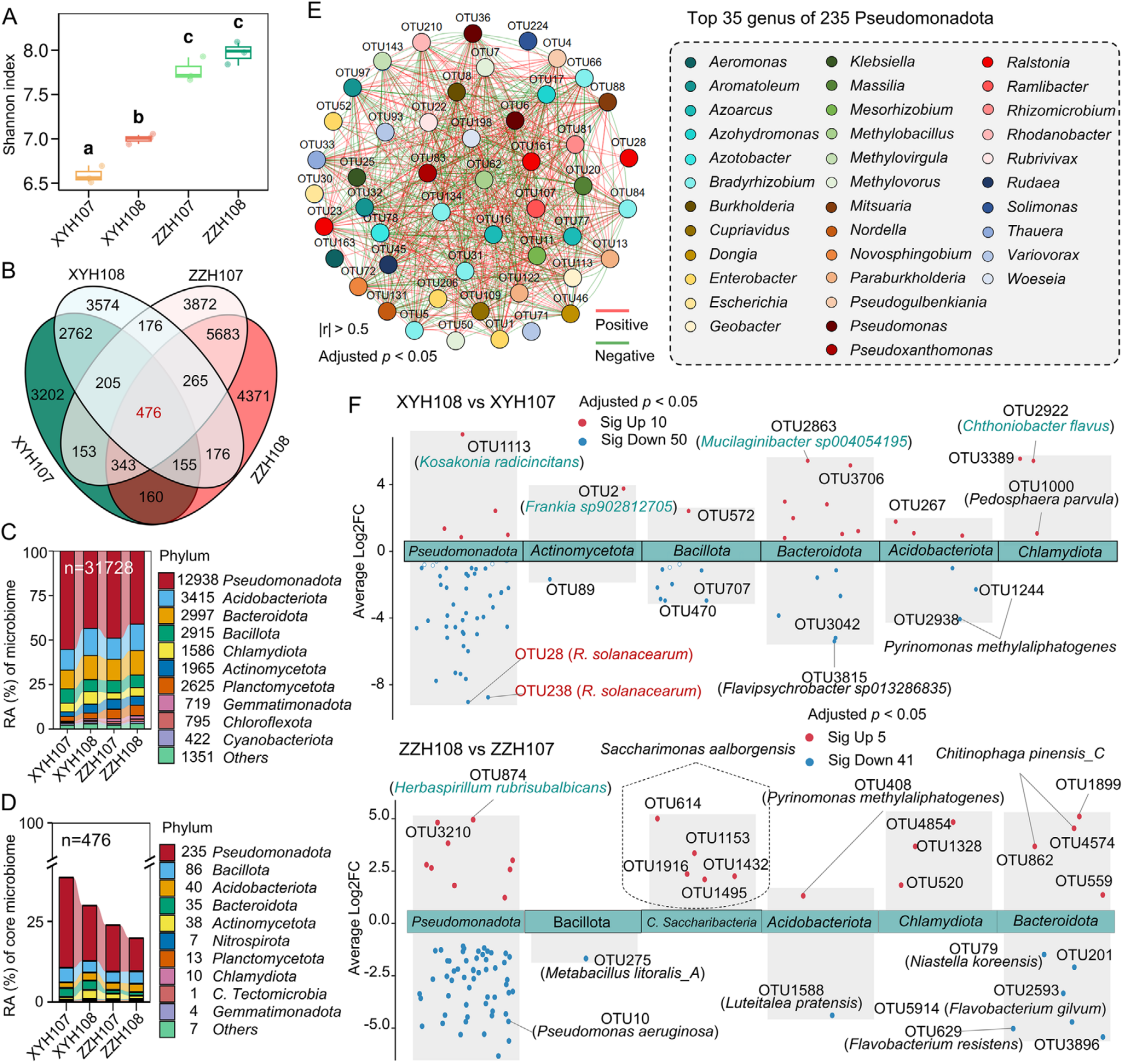
Rhizosphere bacterial diversity and composition of susceptible (H107) and resistant (H108) peanut genotypes under bacterial wilt pressure. (A) Alpha diversity (Shannon index) of rhizosphere bacterial communities. Boxplots indicate median (middle line), percentiles (box), and maximum and minimum values (whiskers), and individual points represent biologically independent replicates (n = 3 replicates). Different letters (a–c) indicate significant differences among treatments (Tukey's test, *p* < 0.05). (B) Total operational taxonomic units (OTUs) (31 728) identified in all samples. (C) Relative abundance (RA) of the top 10 dominant phyla in each sample (n = 3, Tukey's test, *p* < 0.05). (D) RA (%) of the top 10 dominant phyla in the 476 core OTUs identified shared across all samples. For (C‐D), statistically significant differences between the treatments were evaluated by Tukey's test (*p* < 0.05). (E) Co‐occurrence network of the top 35 genera in the core 235 *Pseudomonadota* OTUs. Edges represent strong Spearman correlations (|r| > 0.5): red, positive; cyan, negative. Solid edges indicate statistically significant correlations (two‑sided Student's t‑test, *p* < 0.05). (F) Differential abundance of bacterial species potentially involved in wilt suppression. Points show the average log_2_ fold change (FC) of species with significantly different relative abundance between treatments (Tukey's test, adjusted *p* < 0.05, n = 3). *R. solanacearum* is labeled in red; reported beneficial taxa are labeled in cyan. Red labels denote *R. solanacearum*, and cyan labels denote reported beneficial taxa.

To understand plant‐bacteriome interactions in bacterial wilt resistance, we performed 16S rRNA‐amplicon sequencing of rhizosphere communities from resistant (H108) and susceptible (H107) genotypes with or without *R. solanacearum* infection. While the mock control of the resistant and susceptible genotypes showed similar community profiles, *R. solanacearum*‐infected samples (XYH108, XYH107) exhibited pronounced separation (PERMANOVA, R^2^ = 0.78, *p* = 0.001) (Figure S1A). Across treatments, 903, 923, 1188, and 1171 annotated species were identified in XYH107, XYH108, ZZH107, and ZZH108, respectively (Table S1). Pathogen infection significantly reduced Shannon, Chao 1, Abundance‐based Coverage Estimator (ACE), and Simpson alpha (*α*) diversity indexes in both genotypes compared to controls (3 individual replicates, Tukey's test, *p* < 0.05) (Figure 2A; Figure S1B, C), indicating a loss of both species richness and evenness in the rhizosphere microbiome. Notably, the resistant genotype maintained higher alpha diversity than the susceptible genotype after the *R. solanacearum*‐inoculation (3 individual replicates, Tukey's test, *p* < 0.05), although the Chao1 index and ACE index of the resistant genotype did not differ significantly from the susceptible genotype (3 individual replicates, Tukey's test, *p* > 0.05) (Figure 2A; Figure S1B). These results suggest that the resistant genotype H108 maintains higher community evenness (Shannon and Simpson indices) under pathogen pressure, although the total species richness (Chao1 and ACE indices) was similarly reduced in both genotypes compared to mock controls.

### 2.2 Parsing of the Relationship Between the Pathogen and Beneficial Bacteria

A total of 31 728 operational taxonomic units (OTUs) were identified across all samples (Figure 2B; Table S2), and they were dominated by *Pseudomonadota*, *Bacteroidota*, *Acidobacteriota*, *Bacillota*, and *Actinomycetota*, collectively comprising more than 85% of relative abundance (RA) (Figure 2C; Figure S1C). Notably, RA of the *Pseudomonadota* in the susceptible genotype (XYH107) is higher than that of the resistant genotype (XYH108) under BW infection, while that of *Bacteroidota*, *Acidobacteriota*, and *Bacillota* in XYH108 was higher than that of XYH107 (Figure 2C; Figure S1C). Relative abundance of *Actinomycetota* displayed strong geographical patterns, being more abundant in XYH108 than XYH107, but more abundant in ZZH107 than ZZH108 (Figure 2C). Among the 476 OTUs shared across all samples, defined as core OTUs (Table S3), dominant phyla (including *Pseudomonadota spp*., *Bacillota spp*., *Acidobacteriota spp*., *Bacteroidota spp*., and *Actinomycetota spp*.) remained consistent (Figure 2D).

**FIGURE 3:**
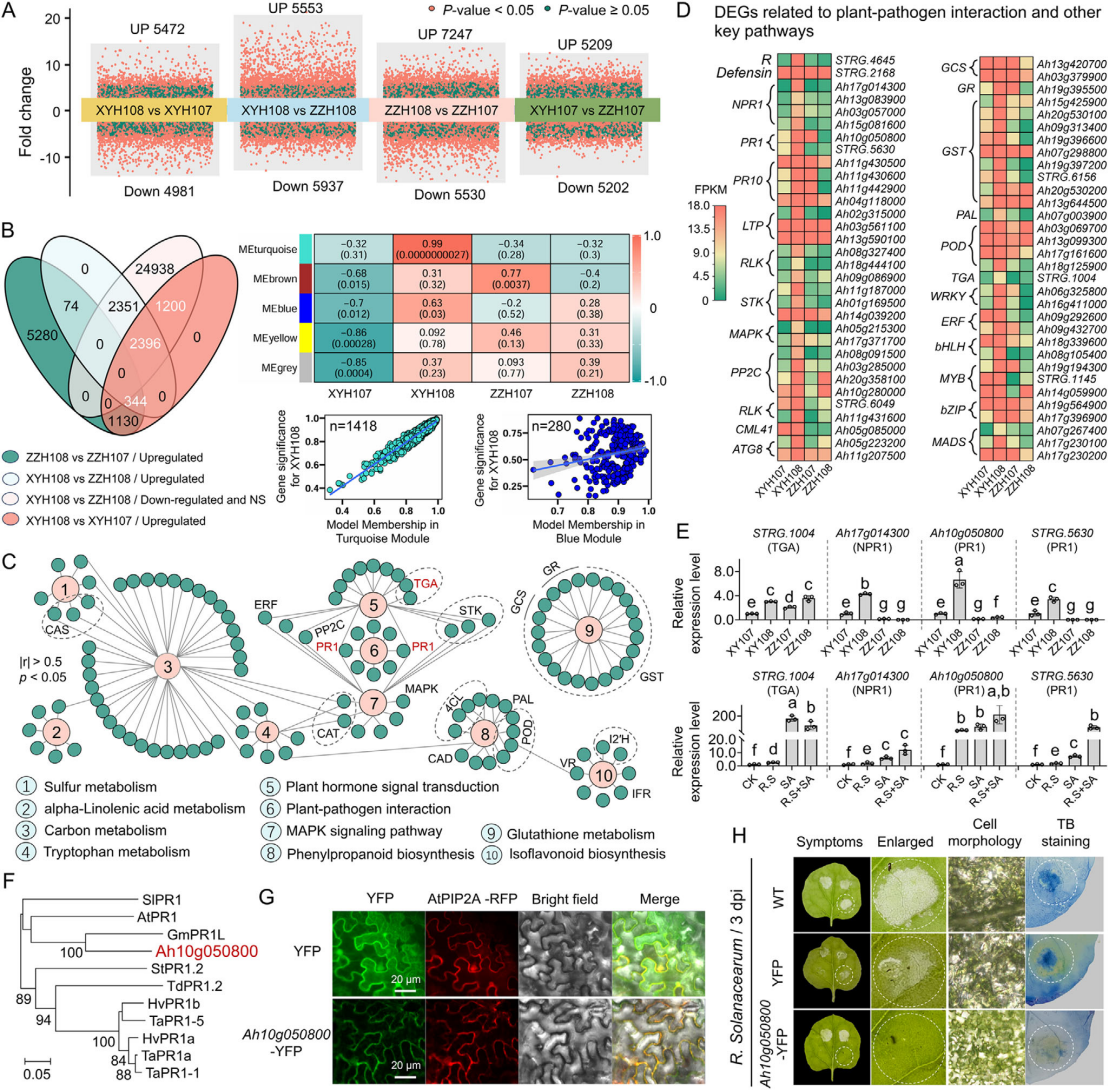
Identification of key pathways and genes associated with H108 resistance against BW. (A) Differentially expressed genes (DEGs) were identified from transcriptomes of XYH107, XYH108, ZZH107, and ZZH108. (B) Selection of core DEGs correlated with the BW resistance in XYH108 (|r| > 0.5, *p* < 0.05). (C) Pathway enrichment and co‐occurrence network of the 1698 core DEGs (|r| > 0.5, *p* < 0.05). (D) Expression profiles of DEGs related to plant‐pathogen interaction and other key pathways. Colors of the mosaics indicate gene expression level values ranging from 0–18.0 (average value of 3 individual replicates). (E) Expression analysis of SAR‐related DEGs in response to *R. solanacearum* infection and exogenous SA. CK and R. S indicate mock‐control and *R. solanacearum*‐infection, respectively. Data are shown as means ± SD, and different letters (a‐f) indicate significant differences among treatments (n = 3 biologically independent samples, Tukey's test, *p* < 0.05). (F) Phylogenetic analysis of Ah10g050800 and other PR1 homologous proteins. The phylogenetic tree was constructed using the neighbor‐joining method with 1000 bootstrap values indicated. (G) Subcellular localization analysis of the Ah10g050800 protein in tobacco leaves. (H) Resistance assessment of transient overexpression of Ah10g050800 against *R. solanacearum*.

To explore the relationships among the species within the microbial communities, a cross‑phyla co‐occurrence network was constructed for the 77 core OTUs with significantly different RA between the resistance and susceptible genotypes (absolute fold change (FC) > 4.0) (Figure S2; Table S4). *Pseudomonadota* taxa occupied approximately half of the OTUs in the network, revealing its predominant role in the peanut‐microbe‐pathogen interaction (Figure S2). Therefore, a co‐occurrence network was constructed for the top 35 genera of the 235 *Pseudomonadota* OTUs (Figure 2E). The *R. solanacearum* (OTU28) was co‐present in the network with reported beneficial taxa, such as *Bradyrhizobium* (OTU31, OTU66, OTU84, OTU134) and *Pseudomonas spp*. (OTU6, OTU36) [49, 50], which suggests a potential interplay between pathogen invasion and the recruitment of beneficial taxa, possibly driven by the host plant under disease pressure.

Analysis of dominant species (bacterial species that probably play crucial roles in bacterial wilt suppression in the peanut rhizosphere) further highlighted distinct microbial signatures between genotypes and locations. Under the *R. solanacearum* infection, the relative abundance of the *R. solanacearum* (OTU28 and OTU238) in the rhizosphere soil of XYH107 was approximately 40‐fold higher (log2FC = 5.3) than in XYH108 (Figure 2F). Meanwhile, beneficial species including *Kosakonia radicincitans* (OTU1113) [51], *Frankia sp902812705* (OTU2) [52], *Mucilaginibacter sp004054195* (OTU2863) [53], *Chthoniobacter flavus* (OTU2922) [54], *Bradyrhizobium sp000938255* (OTU5) [49], *Solibacter usitatus* (OTU159) [55], *Rhizomicrobium sp013149165* (OTU81, OTU2200) [56], and *Mesorhizobium sp003952425* (OTU11, OTU664) [57], were highly enriched in the resistance genotype (XYH108) (Figure 2F; Figure S1D). In mock controls, the susceptible genotype (ZZH107) showed enrichment of beneficial bacterium species including the *Pseudomonas aeruginosa* (OTU10) is significantly enriched in ZZH107 (n = 3, Tukey's test, adjusted *p* < 0.05), whereas ZZH108 was enriched in the beneficial bacterium *Herbaspirillum rubrisubalbicans* (OTU874) (n = 3, Tukey's test, adjusted *p* < 0.05) (Figure 2F; Figure S1D) [50, 58]. These results collectively indicate that resistant genotype tends to suppress *R. solanacearum* abundance in the rhizosphere, at least in part by recruiting beneficial microbial taxa that contribute to its defensive capacity.

Functional annotation analysis revealed that the average proportions of bacterial groups associated with chemoheterotrophy, ureolysis, and nitrogen fixation were significantly more abundant in the resistance genotype (n = 3, Tukey's test, *p* < 0.05), with minimal differences found between ZZH108 and ZZH107 (Figure S3). The functional potential of the rhizosphere microbiome, particularly for processes involved in carbon and nitrogen metabolism, was significantly greater in the resistant genotype XYH108, indicating that the association between a disease‐resistant phenotype and a distinct rhizosphere microbial functional profile is highly genotype‐specific.

### 2.3 Exploring of Dominant Pathways Involved in the BW Resistance

To investigate the genetic resistance of H108 against BW, we performed transcriptome sequencing using roots collected from XYH107, XYH108, ZZH107, and ZZH108 at 70 dpi. A total of 10 453, 11 490, 12 777, and 10 411 differentially expressed genes (DEGs, including both up‐ and down‐regulated genes) were identified in the XYH108 versus XYH107, XYH108 versus ZZH108, ZZH108 versus ZZH107, and XYH107 versus ZZH107 groups (Figure 3A). Pathway enrichment analysis indicated that amino acid metabolism, plant defense response, mitogen‐activated protein kinase (MAPK) signaling, and phytohormone signaling pathways were involved in the resistance against BW (Figures S4 and S5).

To identify core genes specifically associated with resistance in H108, we performed co‐expression analysis on 3940 DEGs that were significantly up‐regulated (n = 3 biologically independent samples, Tukey's test, *p* < 0.05) in XYH108 versus XYH107 but not in ZZH108 versus ZZH107 (Figure 3B; Table S5). The turquoise and blue modules showed strong positive correlations with the BW resistance in XYH108 (r > 0.5, *p* < 0.05) (Figure 3B), and contained 1418 and 280 DEGs, respectively (Table S6). These 1698 core DEGs identified in the turquoise and blue modules through co‐expression analysis were mainly enriched in carbon metabolism, tryptophan metabolism, plant hormone signal transduction, plant‐pathogen interactions, MAPK signaling, phenylpropanoid biosynthesis, and glutathione (GSH) metabolism (|r| > 0.5, adjusted *p* < 0.05) (Figure 3C). Notably, genes encoding TGA transcription factor (TGA), nonexpressor of pathogenesis‐related 1 (NPR1) and pathogenesis‐related 1 (PR1) proteins, key components of the SA‐signaling pathway, were highly represented in the BW resistance network (Figure 3C). Further expression analysis showed that many PR genes [59] and other defense‐related genes were only significantly up‐regulated (average value of three individual replicates, Tukey's test, *p* < 0.05) in the XYH108, but were suppressed in the XYH107, ZZH107, and ZZH108 (Figure 3D; Figure S6). These findings suggest that the SA‐dependent SAR is likely a central mechanism involved in peanut resistance against BW.

### 2.4 Identification of the SA‐Dependent SAR in Peanut Resistance Against BW

To validate the involvement of the SA‐dependent SAR, we examined the expression of four SAR‐marker genes, including the *TGA* (*STRG.1004*), *NPR1* (*Ah17g014300*), and *PR1* (*Ah10g050800* and *STRG.5630*) genes, across all samples using qRT‐PCR (Figure 3E; Table S7). All four genes were significantly up‐regulated (n = 3 biologically independent samples, Tukey's test, *p* < 0.05) in XYH108 following *R. solanacearum* infection (Figure 3E), which is consistent with the transcriptome data. Their expressions were also significantly induced (n = 3 biologically independent samples, Tukey's test, *p* < 0.05) by both *R. solanacearum* and SA in H108 seedlings (Figure 3E). We additionally profiled genes encoding a serine/threonine kinase (STK, *Ah11g187000*), a GSH reductase (GR, *Ah19g395500*), a GSH‐S‐transferase (GST, *Ah19g396600*), and a PR10 protein (*Ah11g430600*) (Table S6). The STK gene *Ah11g187000* and the two GSH metabolism‐related genes (*Ah19g396600* and *Ah19g396600*) showed strong induction under both treatments (n = 3 biologically independent samples, Tukey's test, *p* < 0.05), indicating that GSH metabolism operates alongside SAR to enhance BW resistance (Figure S7).

Based on the expression analysis, *Ah10g050800* (*PR1*) was selected for functional characterization of SAR in BW resistance. A neighbor‐joining phylogenetic tree was constructed using the full‐length amino acid sequence of Ah10g050800 together with ten homologous PR1 proteins from diverse plant species (Table S8). These PR1 proteins were clustered into two major groups, with Ah10g050800 showing the closest genetic relatedness to the GmPR1L in soybean, the AtPR1 in *Arabidopsis thaliana*, and SlPR1 in tomato (Figure 3F). To further examine its function, *Ah10g050800* was cloned into the pCombia1300‐yellow fluorescent protein (YFP) binary vector. Subcellular localization analysis revealed that the Ah10g050800‐YFP fusion protein predominantly accumulated on the plasma membrane in tobacco leaf cells, whereas free YFP distributed throughout the cell (Figure 3G). The resistance of *Ah10g050800* against BW was then evaluated using our previously established transient expression‐based resistance gene identification system [15]. Tobacco leaves overexpressing Ah10g050800–YFP were inoculated with *R. solanacearum*, and no lesions formed, whereas the control exhibited pronounced mesophyll cell death consistent with trypan blue staining (Figure 3H). Collectively, these results demonstrate that Ah10g050800 overexpression enhances resistance to *R. solanacearum*, suggesting that SA‐dependent SAR might contribute to BW resistance in peanut.

### 2.5 Mining of Rhizosphere Metabolites Linking the Beneficial Bacteria and the SAR

**FIGURE 4:**
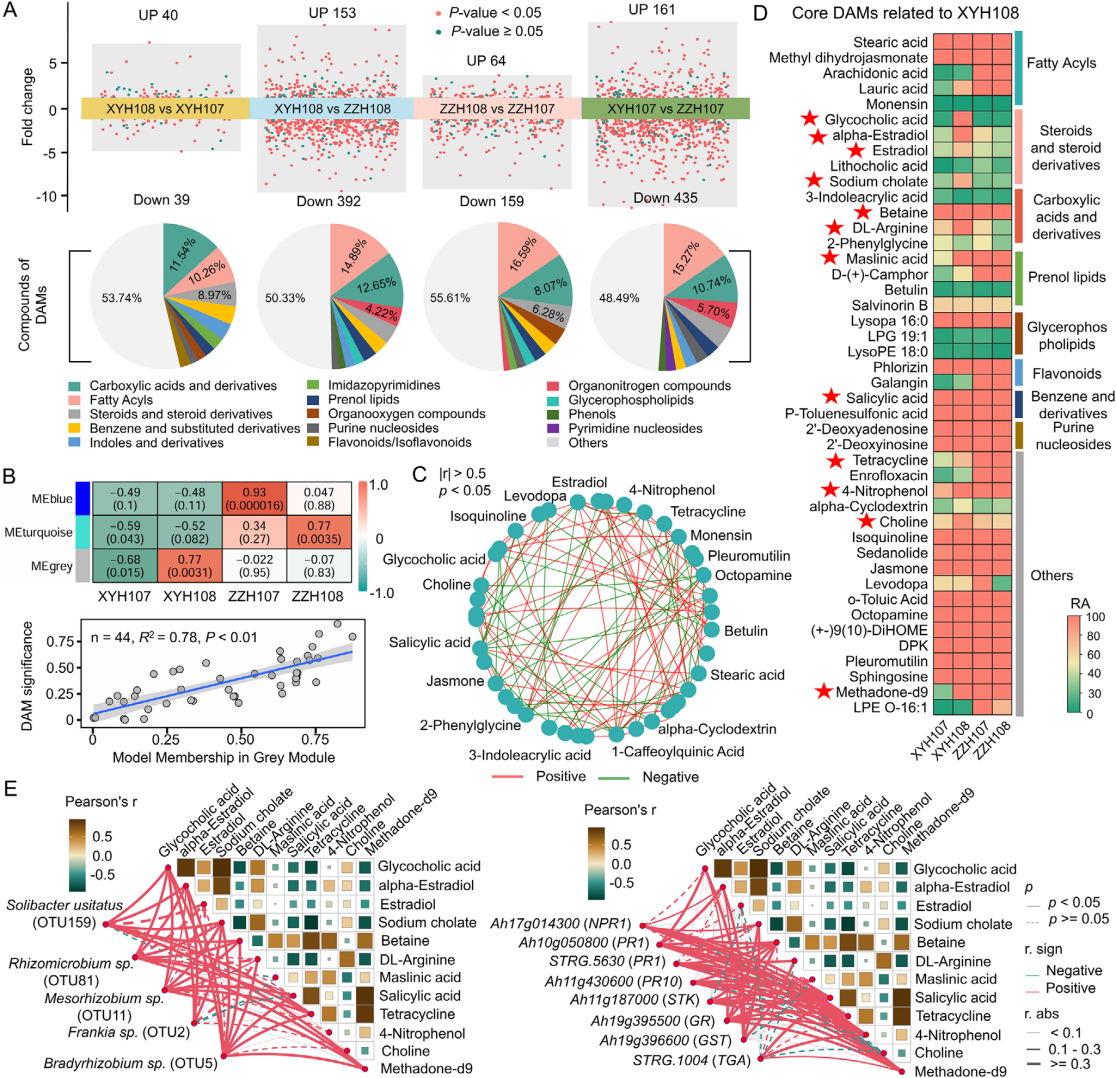
Metabolite profiling of peanut rhizosphere soils of H107 and H108 in from diseased and healthy fields. (A) Differentially accumulated metabolites (DAMs) were identified across the four samples. (B) Weighted Gene Co‐expression Network Analysis (WGCNA) of the 1085 DAMs; the grey module (44 core DAMs) significantly correlates with the suppression of *R. solanacearum* in the XYH108 rhizosphere soil (r = 0.77, *p* < 0.05). (C) Co‐occurrence network of the 44 core DAMs. The network was constructed according to Spearman's correlation coefficients (r), and r < ‐0.5 or r > 0.5 mean negative (cyan lines) or positive (red lines) correlation, respectively. Solid lines indicate significant correlation determined by two‐sided Student's t‐test (*p* < 0.05). (D) RA of the 44 core DAMs detected across the four samples. Red stars indicate the selected 12 DAMs. (E) Pearson's correlation analysis of the 12 key DAMs with the beneficial bacteria and key defense‐related genes. Correlation pairs of *p* < 0.05 and absolute r ≥ 0.3 are considered to be statistically significant correlated, and r > 0 means positive correlation (red lines) while r < 0 means negative correlation (cyan lines).

Given the central role of rhizosphere metabolites in shaping the microbial community, we profiled metabolite changes in peanut rhizosphere soil. To mine key rhizosphere metabolites mediating the interaction between the BW‐resistance and rhizosphere bacteriome, rhizosphere soil was subjected to untargeted liquid chromatography mass spectrometer (LC‐MS) analysis. A total of 1085 compounds were detected across all samples, dominated by lipids, organic acids, and heterocyclic compounds (Table S9). The major categories included lipids and lipid‐like molecules (26.64%), organic acids and derivatives (13.27%), organ‐heterocyclic compounds (8.76%), benzenoids (5.25%), and phenylpropanoids/polyketides (3.96%) (Table S9). In total, 79 and 223 differentially accumulated metabolites (DAMs) were identified in the two comparisons XYH108 versus (vs.) XYH107 and ZZH108 vs. ZZH107, respectively (Figure 4A). These DAMs varied markedly in both abundance and chemical class. Among the 79 DAMs between XYH108 and XYH107, the most enriched categories were carboxylic acids and derivatives (11.54%), fatty acyls (10.26%), and steroids/steroid derivatives (8.97%) (Figure 4A). In contrast, the top three classes in the ZZH107 vs. ZZH108 comparison were fatty acyls (14.89%), carboxylic acids and derivatives (12.65%), and organooxygen compounds (4.22%) (Figure 4A). Kyoto Encyclopedia of Genes and Genomes (KEGG) enrichment analysis showed that these DAMs were primarily associated with metabolism of amino acids, including (such as alanine, aspartate, valine, leucine, and tryptophan) and riboflavin, and biosynthesis of antibiotics (including neomycin, kanamycin, and gentamicin) (Figure S8). DAMs distinguishing the resistance from susceptible genotypes were consistently enriched in metabolism of vitamins (including riboflavin, biotin, and retinol) and amino acids, both under *R. solanacearum* infection and mock conditions (ZZH108 vs. ZZH107 and XYH108 vs. XYH107) (Figure S8). These findings suggest that amino acid, antibiotic, and vitamin metabolic pathways are consistent with peanuts’ suppression of *R. solanacearum*.

To pinpoint keystone metabolites involved in BW suppression in peanut rhizosphere soil, we performed WGCNA using the 1085 detected metabolites. Forty‐four core DAMs from the grey module showed strong positive correlations with the BW resistance in XYH108 (r = 0.77, *p* < 0.05) and were selected for further analysis (Figure 4B; Table S10). These DAMs are mainly composed of fatty acyls (11.36%), steroids/steroid derivatives (11.36%), carboxylic acids and derivatives (9.09%), prenol lipids (9.09%), and glycerophospholipids (6.82%) (Table S10). Notably, well‐known metabolites conferring suppression of soil‐borne pathogens, such as the protective betaine (BTA), defensive‐hormone SA, and the antibiotics tetracycline (TC), occupied central positions in the co‐occurrence network (Figure 4C). Overall, the 44 core DAMs were significantly up‐regulated (Student's *t*‐test, *p* < 0.05) in the rhizosphere soil of the resistance genotype under *R. solanacearum* infection, but were down‐regulated or unchanged between the resistance and susceptible genotypes under mock conditions (Figure 4D). It indicated that those core rhizosphere metabolites are associated with the suppression of *R. solanacearum*.

According to the relative abundance patterns across of the 44 DAMs across the four groups, 12 key rhizosphere metabolites, including glycocholic acid, alpha‐estradiol, estradiol, sodium cholate, BTA, DL‐arginine (Arg), maslinic acid, SA, TC, 4‐nitrophenol, choline, and methadone‐d9, were selected for Pearson's correlation analyses with the relative abundance of the *R. solanacearum* and beneficial bacterial species (Figure 4E). Except for maslinic acid and 4‐nitrophenol, the relative abundances of the remaining 10 metabolites were negatively correlated (|r| ≥ 0.3, *p* < 0.05) with the relative abundance of *R. solanacearum* (Figure S9; Table S11). The relative abundances of glycocholic acid, alpha‐estradiol, sodium cholate, BTA, Arg, SA, TC, and methadone‐d9 were significantly positively correlated (|r|≥ 0.3, *p* < 0.05) with those of more than one of the beneficial bacterial species including *Solibacter usitatus* (OTU159) [55], *Rhizomicrobium sp013149165* (OTU81) [56], *Mesorhizobium sp003952425* (OTU11) [57], *Frankia sp902812705* (OTU2) [52], and *Bradyrhizobium sp000938255* (OTU5) (Figure 4E; Table S12) [49]. Additionally, the relative abundance of glycocholic acid, sodium cholate, BTA, Arg, SA, TC, 4‐Nitrophenol, and methadone‐d9 was positively correlated (|r|≥ 0.3, *p* < 0.05) with the expression levels of defense‐related genes, such as *Ah17g014300* (*NPR1*), *Ah10g050800* (*PR1*), and *STRG.5630* (*PR1*) and *Ah11g430600* (*PR10*) (Figure 4E; Table S13). Together, these results illustrate the probable involvement of those key rhizosphere metabolites in the interaction between beneficial bacteria and the defense response.

### 2.6 Development of the Rhizosphere Metabolite Prebiotics

**FIGURE 5:**
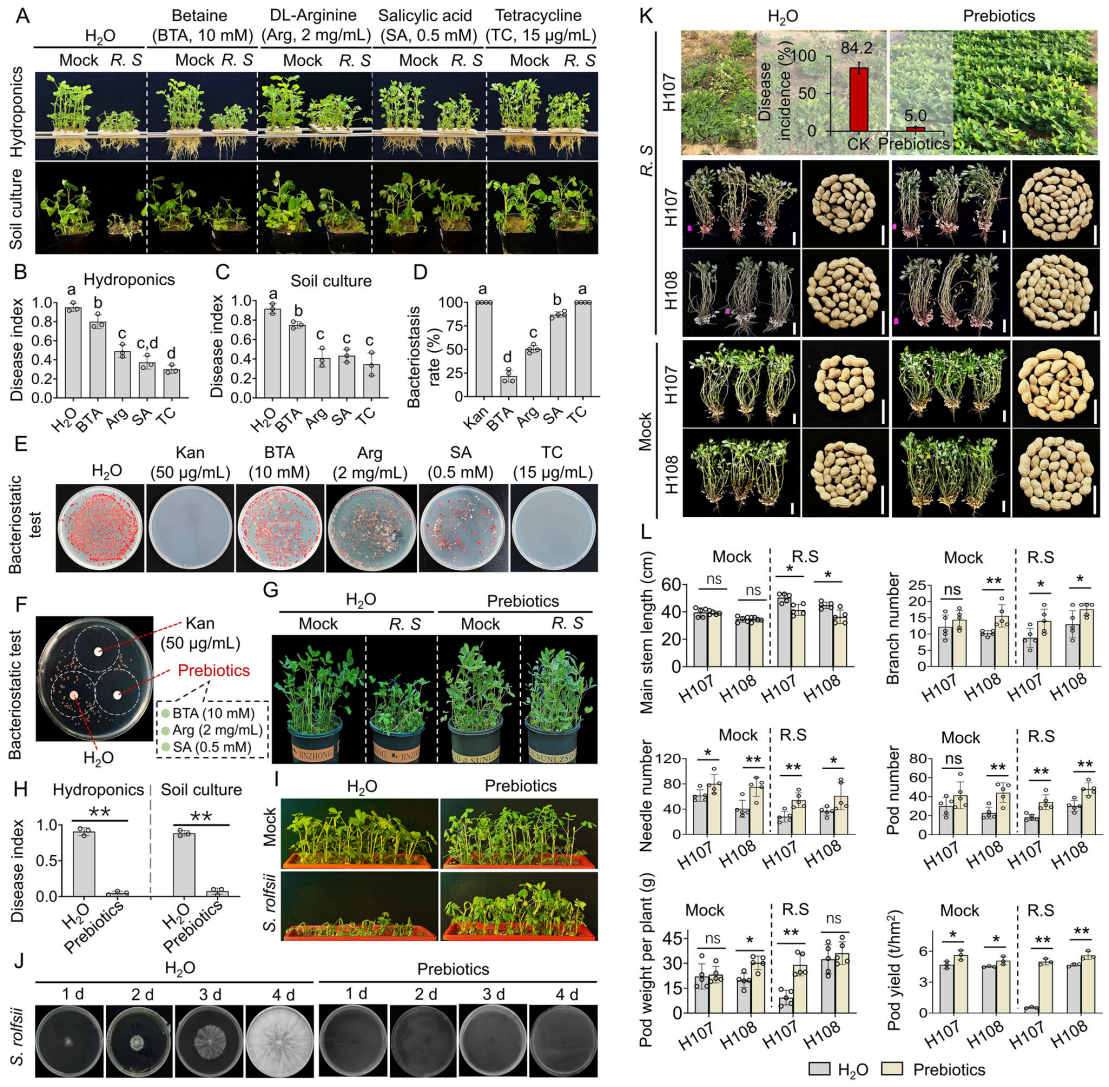
Development and field application of the prebiotics. (A) Schematic of hydroponic and soil assays testing the suppression of *R. solanacearum* infection by root and soil‐drenching application of the four commercial metabolites. Mock and *R. S* indicate mock‐control and *R. solanacearum*‐infection, respectively. (B and C) Effects of the metabolites on the disease index of H107 under *R. solanacearum* infection in the hydroponics and soil‐culture experiments. (D and E) In vitro antibacterial tests of the four metabolites against *R. solanacearum*. For (B–D), data are shown as means ± SD, and different letters (a–d) indicate significant differences among treatments with 3 biologically independent samples (Tukey's test, *p* < 0.05). (F) Development and in vitro antibacterial test of the prebiotics (containing 10‐mm BTA, 2‐mg/mL Arg, and 0.5‐mm SA) against *R. solanacearum*. (G) Soil‐drenching application of the prebiotics to H107 plants under *R. solanacearum* infection. (H) Effects of the root and soil‐drenching application of the prebiotics to the disease indexes of hydroponics and soil‐culture H107 plants under *R. solanacearum* infection. Each data point represents a biologically independent replicate, and data are shown as mean ± SD (n = 3). Variations with significant difference among treatments were determined by Student's *t*‐test, and a single asterisk (*) represents *p* < 0.05 and double asterisks (**) indicate *p* < 0.01. (I) Effects of the prebiotics’ application on the resistance of H107 to the fungal disease stem rot caused by the *Sclerotium rolfsii*. (J) In vitro antibacterial tests of the prebiotics on the growth of *S. rolfsii*. (K) Performance of the prebiotics in enhancing peanut resistance against BW. Disease incidences are shown as mean ± SD (n = 3), and ** indicates a significant difference (Student's *t*‐test, *p* < 0.01). Bars indicate 5 cm. (L) Effects of the prebiotics on the growth and yields of peanuts in the diseased and healthy field. Each data point represents a biologically independent replicate, and data are shown as mean ± SD (n = 5). Variations with significant difference among treatments were determined by Student's *t*‐test and indicated with * (*p* < 0.05) and ** (*p* < 0.01). ns indicates no significant difference.

Our comprehensive metabolomic and microbiome analyses enabled the development of rhizosphere metabolite prebiotics for efficient management of BW in peanut production. The four commercially available metabolite compounds, including the BTA, Arg, SA, and TC, were selected from the 12 key DAMs for functional testing. Three‐week‐old H107 seedlings were inoculated with *R. solanacearum* and subsequently treated with 10‐mm BTA, 2‐mg/mL Arg, 0.5‐mm SA, or 15‐µg/mL TC, respectively (Figure 5A). The strong antibiotic TC was used as an internal positive control. Hydroponic assays (root application) and soil‐pot experiments (Soil‐drenching applications) were conducted simultaneously. The disease incidence of peanut BW in each treatment was investigated and statistically analyzed at 14 dpi. None of the metabolites affected the growth of mock‐treated plants, but all four significantly reduced the disease incidence of BW in inoculated H107 seedlings (n = 3, Tukey's test, *p* < 0.05) (Figure 5A–C). Following root application, the disease index decreased from 0.95 to 0.80, 0.49, 0.37, and 0.30 after treatment with BTA, Arg, SA, and TC, respectively (n = 3, Tukey's test, *p* < 0.05) (Figure 5B). Soil‐drenching application similarly reduced disease indices from 0.91 to 0.75, 0.41, 0.43, and 0.34 (n = 3, Tukey's test, *p* < 0.05), respectively (Figure 5C). These findings demonstrate that exogenous application of these rhizosphere metabolites might enhance BW resistance regardless of delivery method.

To determine their roles in suppressing *R. solanacearum*, we evaluated the antibacterial activities of BTA, Arg, SA, and TC against *R. solanacearum* in vitro. We found that 50‐µg/mL kanamycin (Kan, used as a positive control) and 15‐µg/mL TC (used as an internal control) completely inhibit the growth of *R. solanacearum*, while 10‐mm BTA, 2‐mg/mL Arg, and 0.5‐mm SA also displayed moderate inhibitory (n = 3, Tukey's test, *p* < 0.05) (Figure 5D,E). Specifically, the bacteriostasis rate for BTA, Arg, SA, and TC against *R. solanacearum* were 21.71%, 50.47%, 86.90%, and 100.00%, respectively.

Prioritizing ecological safety, field practicability, and cost‐efficiency, solutions containing 10‐mm BTA, 2‐mg/mL Arg, and 0.5‐mm SA were combined in equal proportions to formulate the prebiotics, which exhibited enhanced antibacterial effect against *R. solanacearum* (Figure 5F). To assess its efficacy in planta, the prebiotics were applied to the susceptible H107 plants under *R. solanacearum*‐infection via root and soil‐drenching application. Both application methods markedly reduced BW incidence without affecting plant growth (Figure 5G; Figure S10A). Disease indexes dropped from 0.91 and 0.88 in controls to 0.05 and 0.08 for root and soil‐drenching applications (n = 3, Student's *t*‐test, *p* < 0.05), respectively (Figure 5G,H). Additionally, application of the prebiotics significantly limited *R. solanacearum* colonization in peanut roots (n = 3, Student's *t*‐test, *p* < 0.05) (Figure S10B). Beyond bacterial wilt, the prebiotics strongly suppressed peanut stem rot, one of the most devastating fungal diseases caused by *Sclerotium rolfsii* (Figure 5I; Figure S10C). The sclerotium germination and the hyphal growth were completely inhibited by the prebiotics (Figure 5J; Figure S10D). Collectively, the natural prebiotics show promising potential for efficient, environmentally compatible, and cost‐effective management of peanut diseases under the tested conditions.

### 2.7 Field Application of the Rhizosphere Metabolite Prebiotics

To test its field utility, the effects of the prebiotics on peanut resistance against BW were investigated. The seeds of the resistance (H108) and susceptible (H107) genotypes were soaked overnight in the prebiotic solution prior to sowing in both a BW‐diseased field and an *R. solanacearum*‐free field. Two additional soil‐drenching applications were conducted at 15‐and 30‐days post seedling emergence. With the application of the prebiotics, the disease incidence decreased from 84.2% to 5.0% (approximately 94%) in H107 (n = 5 biologically independent samples, Student's *t*‐test, *p* < 0.05), and from 8.32% to 0.20% (about 98%) in H108 at harvest (n = 5, Student's *t*‐test, *p* < 0.05), respectively (Figure 5K).

Moreover, application of the prebiotics also promoted growth and yield of peanuts both under *R. solanacearum* infection and control conditions (Figure 5K; Figure S11). Although the length of the main stem and branches was significantly decreased by the prebiotics under *R. solanacearum* infection (n = 5, Student's *t*‐test, *p* < 0.05), branch numbers were significantly increased (n = 5, Student's *t*‐test, *p* < 0.05) (Figure 5L; Figure S11). Notably, the pod numbers of H107 and H108 increased by 38.00% to 93.86% by the application of the prebiotics (n = 5, Student's *t*‐test, *p* < 0.05) under *R. solanacearum* infection and mock control with the increasing of pod needle numbers for 29.87–93.57% (n = 5, Student's *t*‐test, *p* < 0.05) (Figure 5L). Full‐pod numbers and pod weight per plant were also increased by the prebiotics (n = 5, Student's *t*‐test, *p* < 0.05), while the weights of hundred pods and seeds were slightly affected (Figure 5L; Figure S11). Ultimately, application of the prebiotics preserved 84.91% of H107 yield under *R. solanacearum* infection (n = 5, Student's *t*‐test, *p* < 0.01), and boosted overall yields of H107 in both cultivars by 12.87‐20.30% under control conditions (n = 5, Student's *t*‐test, *p* < 0.05) (Figure 5L). Those results indicated potential for broad application of the prebiotics in guaranteeing the yield of peanuts under *R. solanacearum* infection.

### 2.8 The Prebiotics Enhance Peanut Resistance via Recruiting Beneficial Microbes

**FIGURE 6:**
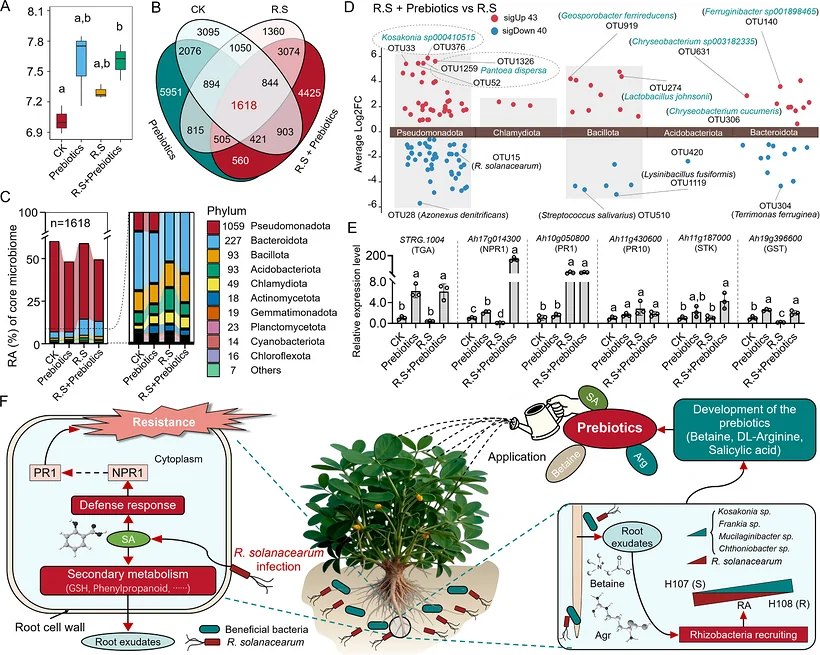
Potential mechanisms of the prebiotics in enhancing peanut resistance against BW. (A) Shannon indices indicated rhizosphere bacterial community diversity of H107 plants in response to the prebiotics’ application under BW infection. Boxplots indicate median (middle line), percentiles (box), and maximum and minimum values (whiskers) (n = 3 individual replicates), and different letters (a‐b) indicate significant differences among treatments (Tukey's test, *p* < 0.05). (B) The 35 170 OTUs identified in the four groups of control, application of the prebiotics (Prebiotics), *R. solanacearum* infection (R.S), and R.S + Prebiotics. (C) RA (%) of the top 10 dominant phyla identified in the 1618 core OTUs of the rhizosphere bacterial communities. (D) Bacterial species with significant differences in the R.S + prebiotics vs the R.S group. Each data point represents the average Log_2_FC of bacterial species with significant differences between different treatments (3 biologically independent samples, Tukey's test, adjusted *p* < 0.05). (E) Effects of the prebiotics on the expressions of key defense‐related genes. Data are shown as means ± SD, and different letters (a–d) indicate significant differences among treatments (n = 3 biologically independent samples, Tukey's test, *p* < 0.05). (F) Schematic model summarizing resistance mechanisms of H108 plants coping with the *R. solanacearum* infection. Black lines with arrows represent promotion, and boxes indicate genes, metabolism processes, or microbiome remodeling.

To investigate mechanisms underlying the prebiotics‐mediated protection, the 16S rRNA profiling was conducted on rhizosphere soils collected 30 days after the final prebiotics application. Pronounced shifts in bacterial community composition were observed between the control and application of the prebiotics (Prebiotics) treatments (*p* < 0.05), while differences between the *R. solanacearum* infected (R.S) and R.S + Prebiotics treatments were subtler (Figure S12A). The Chao 1 and ACE indexes of the prebiotics‐treated peanut rhizosphere soils (namely Prebiotics and R.S + Prebiotics samples) were significantly lower than those of untreated plants (n = 3 individual replicates, Tukey's test, *p* < 0.05), indicating that the estimated total number of species (including rare species) and the overall species richness were decreased with the prebiotics application (Figure S12B). Additionally, the significantly increased Shannon and Simpson indexes (n = 3 individual replicates, Tukey's test, *p* < 0.05) revealed the prebiotics effectively improved the structure of the rhizosphere microbial community, making it more diverse and evenly distributed (Figure 6A; Figure S12B). Meanwhile, more annotated species were identified in the Prebiotics and R.S + Prebiotics samples than those of control and R.S treatments, respectively (Table S14). These results suggested that the prebiotics application reshaped the community structure through reducing the overall number of species and enhancing the evenness, potentially leading to greater functional stability and resilience.

Across all samples, we identified 35 170 OTUs, which were dominated by *Pseudomonadota*, *Bacteroidota*, *Bacillota*, *Acidobacteriota* and *Chlamydiota* (Figure 6B; Figure S12C; Table S15). The 1618 core OTUs were dominant, with the *Pseudomonadota* bacterium accounting for > 65.45% relative abundance (Figure 6C; Table S16). Further functional annotation analysis revealed that the differentially enriched bacterium species are related to chemoheterotrophy, nitrogen fixation, and plant pathogens in the comparison groups of Prebiotics vs Control and R.S + prebiotics vs R.S (Figure S12D). Notably, the relative abundance of various beneficial bacterial species, such as *Kosakonia sp000410515* (OTU33 and OTU376) [51], *Pantoea dispersa* (OTU52, OTU1259 and OTU1326) [60], *Geosporobacter ferrireducens* (OTU919) [61], *Lactobacillus johnsonii* (OTU274) [62], *Pseudomonas aeruginosa* (OTU391) [50], and *Chryseobacterium cucumeris* (OTU306) [63], were significantly increased by the application of the prebiotics (n = 3, Tukey's test, adjusted *p* < 0.05) (Figure 6D; Figure S12E; Figure S13). These results indicated that the prebiotics application promotes recruitment of disease‐suppressive bacteria in the peanut rhizosphere.

Given the role of SA‐dependent SAR in the war of H108 against *R. solanacearum*, we assessed whether the prebiotics modulate host defense response. The roots of peanut plants with the Control, Prebiotics, R.S, and R.S + Prebiotics treatments were collected at 7 days post the first prebiotics foliar application, and expression profiles of the genes *Ah17g014300* (NPR1), *Ah10g050800* (PR1), and *STRG.5630* (PR1) and *STRG.1004* (TGA), as well as the *Ah11g187000* (STK) and Ah19g396600 (GST) were examined by qRT‐PCR. All six genes were all significantly up‐regulated by the prebiotics application when compared with that of control and *R. solanacearum* infected conditions (n = 3 biologically independent samples, Tukey's test, *p* < 0.05) (Figure 6E). These results indicate that the application of the prebiotics enhances both beneficial bacteria recruitment and SA‐dependent SAR, collectively improving peanut resistance to *R. solanacearum* (Figure 6F).

## 3 Discussion

Climate change is intensifying the expansion of soil‐borne pathogens, including *R. solanacearum* and *S. rolfsii*, causing devastating losses [4, 64, 65, 66]. Conventional management of soil‐borne diseases in peanuts typically relies on chemical, cultural, and biological strategies [65, 67, 68]. However, pathogens residing in deep soil layers or colonizing in the xylem, and environmental and residue concerns, limit the efficacy of conventional chemical treatments [69, 70]. Compared with chemical agents, taking advantage of varieties with high resistance is one of the most eco‐friendly and effective means for soil‐borne disease management [65, 67, 71]. This study reveals the defense strategy of the highly BW‐resistant genotype H108. Our findings highlighted that rhizosphere metabolic reprogramming synergizes with systemic immunity to suppress bacterial wilt, a mechanism that integrates plant genetic traits with microbiome ecology (Figure 6F). Crucially, we successfully translated this biological insight into an effective prebiotic formulation, achieving dramatic disease control and yield enhancement in field conditions.

Our results establish that SA‐dependent SAR is activated in the resistant peanut genotype H108 upon *R. solanacearum* challenge, marked by the up‐regulation of NPR1 and PR genes [17, 18, 19, 20]. The cloned SAR‐related gene *Ah10g050800* (*PR1*), which we functionally validated, showed close genetic relationships with cloned resistance genes, including the *GmPR1L*, *AtPR1*, and *SlPR1* (Figure 3F). Thereinto, the *GmPR1L* confers resistance to fungal pathogen *Cercospora sojina* Hara in soybean [72], the well‐known *AtPR1* exhibits broad‐spectrum resistance against a variety of pathogens [73], and the *SlPR1* in tomato confers the resistance against fungal pathogen *Fusarium oxysporum* [74]. This highlights that the application prospects of SAR‐related genes are promising targets for molecular breeding of resistant peanut cultivars. Beyond intrinsic immunity, advances in microbiome research have highlighted microbial inoculants as potential tools for agricultural disease management [75]. Beneficial taxa, such as *Bacillus*, *Chryseobacterium*, *Geosporobacter*, *Lactobacillus*, *Pseudomonas*, *Rubrivivax*, and *Trichoderma* can suppress key soil‐borne pathogens [76, 77, 78, 79]. While such beneficial taxa are promising as microbial inoculants, their establishment in complex soil environments remains a challenge. The resistant peanut genotype H108 demonstrated remarkable capacity in recruiting rhizosphere beneficial microorganisms, including *Kosakonia sp*., *Frankia sp*., *Mucilaginibacter sp*., *Bradyrhizobium sp*., *Rhizomicrobium sp*., and *Mesorhizobium sp*. (Figure 2F; Figure S1D) [49, 51, 52, 53, 56, 57]. Those beneficial bacteria target globally significant soil‐borne pathogens, including *Pythium*, *Phytophthora*, *Fusarium*, *Rhizoctonia*, and *Verticillium*, demonstrating efficacy in lab and field settings [80, 81, 82]. However, exogenous microbial inoculants often struggle to establish dominant populations in complex and dynamic soil environments [83, 84]. Notably, pathogen abundance was substantially lower in the rhizosphere soil of the resistant genotype (Figure 2F). Although our data cannot definitively separate whether microbiome recruitment drives pathogen suppression or is a consequence of reduced pathogen pressure, the strong association suggests a synergistic interaction (Figure 4E). Therefore, the observed reduction in pathogen abundance in the resistant genotype could be driven by host resistance (e.g., SAR), accompanied by the distinct microbiome being a secondary consequence of this altered root environment, or a combined effect where recruited microbes and host resistance synergistically contribute to the suppression [27, 28, 29, 30].

The rhizosphere of the resistance genotype is enriched with specific metabolites that function as chemical signals, effectively recruiting beneficial bacteria [85, 86, 87]. The enrichment of genotype‐specific beneficial microbes in the rhizosphere was accompanied with the activation of SA‐dependent SAR (Figures 2 and 3). Additionally, SA was detected to be higher accumulated in the rhizosphere‐soil metabolites of H108 in response to BW infection (Figure 4). These results are aligned with the recently proposed cry for help paradigm, where plants under threat would reshape their rhizosphere microbiome [27, 28]. While SA‐dependent SAR and the ‘cry for help’ model, wherein plants reshape their rhizosphere microbiome under stress, are established paradigms in plant immunity, their integrated operation and relative contributions in mediating genotype‐specific resistance against soil‐borne *R. solanacearum* in peanut remain largely unexplored. Through multi‐omics association analysis, we provided data‐driven evidence indicating that in the resistant genotypes, the endogenous SA signal, the accumulation of SA in the rhizosphere, the enrichment of beneficial microbial groups, and the inhibition of pathogenic bacteria all exhibit a high degree of statistical correlation, forming a self‐enhancing defense loop (Figure 6F). Apart from SA, betaine and arginine were also identified to be keystone metabolites enhancing beneficial bacteria enrichment and SAR response in peanuts against BW (Figures 4 and 5). Betaine functions as an osmoprotectant and signaling molecule or nutrient source, thereby influencing plant‐microbe interactions [88, 89]. Arginine acts as a nitrogen source and a precursor for crucial signaling molecules, including nitric oxide and polyamines, which are integral to the recruitment of beneficial microbes and the plant immune response [90, 91, 92, 93]. In the present study, we identified and verified a previously unknown key metabolic compound combination with a synergistic effect in the context of peanut resistance to *R. solanacearum*, and the combined effect of the three compounds (prebiotics) is superior to that of any single component (Figure 5), suggesting a complex chemical recruitment signal rather than a single one. All these results indicate crucial roles of keystone metabolites in regulating defense response and in the rhizosphere of the resistant genotype, acting as a biological trigger for peanut coping with *R. solanacearum* infection.

Guided by the elucidated mechanism, we developed the prebiotics, a mixture of rhizosphere keystone metabolites, designed to simultaneously mimic the plant's recruitment signals to create a more favorable niche for beneficial colonizers. Though rhizosphere compounds and concentrations are dynamic, our multi‐omics data revealed potential roles of some compounds in bacterial wilt suppression in the peanut rhizosphere. Key compounds, such as betaine, SA, and arginine, were selected based on our Pearson's correlation analyses and previous reports identifying them in mediating plant‐microbe interactions and stress responses. Prebiotics often inhibit pathogen growth [94], trigger plant immune‐related SAR [95, 96], and selectively promote the growth and activity of beneficial soil microorganisms such as *Bifidobacteria* and *Lactobacillus* [48, 97. 98]. The prebiotics application reduced the disease incidence of BW from 84.2% to 5.0% (Figure 5K,L), which convincingly confirmed the success of this strategy. This formulation addresses the limitations of conventional chemical controls, such as environmental persistence and pathogen resistance [70]. Compared with conventional chemical bactericides, the metabolite‑only prebiotics likely exert lower selection pressure for pathogen resistance, reducing the risk of rapid resistance evolution. However, repeated application over multiple years might still affect non‑target soil microbes, and long‑term ecological consequences remain unknown. Future studies should monitor both resistance dynamics and potential shifts in the resident microbial community. Furthermore, the prebiotics treatment led to a significant yield increase of 12.87% to 20.30% (Figure 5K,L). Although the selected compounds and concentrations do not precisely reflect actual rhizosphere conditions in peanut fields, those compounds and concentrations appeared to effectively guarantee the yield of peanut under BW challenge, as reported in other crops. This outcome is critical as it dispels potential concerns that biocontrol measures might trade resistance for productivity due to energy costs.

Recent evidence suggests that combining prebiotics with beneficial microbial inoculants may synergistically enhance plant immunity and pathogen suppression [47, 48, 97, 98]. Simple delivery methods, such as seed coatings enriched with prebiotic compounds and beneficial microbes, may confer early‐stage disease protection and promote seedling vigor. This offers an eco‐friendly alternative to reducing soil degradation and promoting long‐term agricultural sustainability. By advancing our understanding of plant‐microbe interactions, this study lays the foundation for developing next‐generation agricultural solutions that prioritize environmental health and food security in an unpredictable climate challenging.

## 4 Conclusion

Taken together, our research establishes a direct connection between the rhizosphere microbiome composition and systemic immune activation in peanut plants, revealing a coordinated defense strategy to soil‐borne diseases. To cope with the *R. solanacearum* infection, the resistant peanut genotype reshapes its rhizosphere bacterial community which leads to the enrichment of beneficial bacteria. This external remodeling is coupled with internal SA‐dependent SAR mediated through modulating defense‐related *NPR1* and *PR1*. Keystone rhizosphere metabolites, such as betaine, DL‐arginine, and SA, are associated with enhanced recruitment and immune signaling, thereby creating a feedback loop that reinforces plant immunity. Leveraging these insights, we formulated a peanut‐specific prebiotic, which effectively enhances beneficial microbial recruitment and stimulates SAR. Field application of the prebiotics reduced BW incidence from 84.2% to 5.0% and increased yields by 12.87%‐20.30%. This evidence suggests that the prebiotics may serve as a dual‐purpose solution, integrating “plant protection” and “yield promotion,” thereby enhancing its agricultural application value.

## 5 Experimental Section

### 5.1 Field Cropping of the Resistant and Susceptible Peanut Genotypes

To investigate interactions between rhizosphere soil conditions and genetic resistance of peanut plants under *R. solanacearum* infection, the susceptible (H107) and resistant (H108) peanut cultivars were planted both in the BW‐diseased and *R. solanacearum*‐free soils. For BW‐resistance evaluation, the susceptible and resistant blocks were cropped in the BW‐prevalent field at Xinyang (32°32′N, 112°42′E), south Henan, China. As mock controls, the susceptible (H107) and resistant (H108) genotypes were also planted in the healthy field at Zhengzhou (34°87′N, 113°6′E), north Henan, China. Both in the BW‐infection and mock control treatments, H107 and H108 were planted in the susceptible and resistant blocks in a randomized block design. Totally, 12 blocks were set, thereinto 6 blocks were BW‐infected (3 biological replications), and the remaining 6 blocks were mock control. For the 6 BW‐infection blocks and the 6 mock control blocks, respectively, three of them were planted with H107, and the remaining three blocks were H108. Accordingly, three biological replications were set for each of the four treatments (XYH107, XYH108, ZZH107, and ZZH108). Each block contained 5 rows (2‐m length and 30‐cm space), and 15 healthy peanut seeds were sown in each row (one seed per well with 10‐cm space). Field farming management measures, including watering and fertilizer application, were manually applied as commonly conducted.

For a sufficient incidence rate and the same pathogenic pressure, three‐week‐old peanut seedlings growing in the BW‐diseased field were inoculated with *R. solanacearum* bacterial inoculum. Briefly, the virulent *R. solanacearum* strain isolated from the diseased peanut plants collected from Xinyang was initially cultured on the triphenyl tetrazolium chloride (TTC) agar medium. Following overnight cultivation, fresh red colonies were picked up with a sterilized toothpick and propagated in TTC broth. The bacterial suspension culture was adjusted to approximately 10^8^ colony‐forming units (CFU) / mL (corresponding to an OD_600_ = 0.1). Subsequently, peanut plants were inoculated with *R. solanacearum* by watering with 100‐mL prepared bacterial inoculum per plant. The pathogen inoculation was repeated once again at 7 days post‐first inoculation, and the moist soil was maintained by spraying with water until 80 days post‐sowing. The bacterial wilt symptoms caused by *R. solanacearum* infection, namely, more than half of the peanut leaves appear dehydrated with wilting phenotypes despite remaining green, were daily observed and recorded before harvesting. The final survival rates were obtained by calculating the ratios of surviving peanut plants among the total plants at 80 days post‐sowing.

### 5.2 Collection of Rhizosphere Soil and Root Tissues from Peanut Plants

Rhizosphere soil and root tissues were randomly collected from BW‐diseased peanut plants in XYH107 blocks, and peanut plants in XYH108, ZZH107, and ZZH108 blocks at 70 days post sowing, respectively. Briefly, five peanut plants were randomly selected from each block, and excess soil on the roots was discarded by gently shaking the plants. The remaining soil attached to the surface of roots was collected as rhizosphere soil, and the rhizosphere soil of the five peanut plants was fully mixed into a single composite soil sample. Each composite soil sample was equally divided into two portions, and approximately 5.0‐g soil samples were used for soil DNA extraction and metabolite profiling, respectively. Meanwhile, root tissues of the five peanut plants collected from each block were washed with tap water twice and then cut into 1‐cm pieces. Approximately 2.0‐g root tissues were retained in the RNase‐free centrifuge tubes and surface‐sterilized with 2% sodium hypochlorite solution for 5 min, followed by being washed with sterile water for 3 times. Experimental operations were conducted on ice as far as possible. All samples from the peanut plants with different treatments were collected with three biological replications, and stored at ‐80°C after quick freezing with liquid nitrogen. For sample collecting, rhizosphere soil and root tissues randomly collected from five plants of each block were pooled into a single composite sample, and three composite samples for each of the four treatments were used for subsequent analysis.

### 5.3 Soil DNA Extraction and 16S rRNA Bacterial Community Diversity Analysis

Total soil DNA was extracted from 0.5‐g rhizosphere soil of each sample using the Power Soil DNA Isolation Kit (Qiagen, Hilden, Germany). The obtained DNA was evaluated with gel electrophoresis and was quantified using a Nanodrop 2000c spectrophotometer (Nanodrop, Thermo Fisher Scientific, Waltham, USA). The primer pair 27F (AGRGTTYGATYMTGGCTCAG) and 1492R (RGYTACCTTGTTACGACTT) was used to amplify the V1‐V9 hypervariable region of the bacteria 16S ribosomal RNA gene. The 50‐µL PCR reaction mixtures contained 3.0‐µL DNA (20 ng/µL), 1.0‐µL each primer (10 µm), 20.0‐µL ddH_2_O, and 25.0‐µL 2 × Premix Taq (Takara, Dalian, China). The specific fragment of the 16S ribosomal RNA gene was amplified using a Bio‐Rad S1000 system (Bio‐Rad Laboratory, CA, USA) with the following cycles: 95°C for 2 min, followed by 27 cycles at 95°C for 30 s, 55°C for 30 s, and 72°C for 60 s, and a final extension at 72°C for 5 min. The PCR products were checked on agarose gels (2.0%), and the clear bands with a length of 1400–1500 bp were extracted and purified using the AxyPrep DNA Gel Extraction Kit (Axygen Biosciences, Union City, CA, U.S.).

Sequencing DNA libraries were prepared from the purified DNA using the NEBNext UltraTM DNA Library Prep Kit for Illumina (New England Biolabs, USA). All amplicon sequencing was performed on a single PacBio Sequel II cell by Shanghai Biozeron Biotechnology Co., Ltd. (Shanghai, China). Raw reads were processed using the SMRT Link Analysis software (version 9.0) and the SMRT Portal to obtain clean and high‐quality reads (< 800 or > 2500 bp). Barcode and primer sequences, and sequences that contained 10 consecutive identical bases, were further removed. Using the UPARSE (version 7.1), OTUs were clustered with 98.65% similarity cutoff, which was determined through a data‐driven optimization process to maximize biological relevance and accuracy. The Chao1, ACE, Shannon, and Simpson diversity indexes were revealed by the rarefaction analysis based on the Mothur (v.1.21.1), and the beta diversity analysis was performed using the UniFrac to compare the results of the principal component analysis (PCA).

To determine the relationships between microbiota and treatments, the Spearman's correlation coefficients were assessed. One‐way analysis of variance (ANOVA) was performed to assess the statistically significant difference (*p* < 0.05) of diversity indexes between samples with different treatments. The online “Draw Venn Diagram” tool was used to generate Venn diagrams to analyze overlapped and unique OTUs among different samples. The co‐occurrence network was constructed on the online tool of Majorbio Cloud Platform (https://www.majorbio.com/tools), according to the Spearman's correlation coefficients (r). A correlation relationship with a r < ‐0.5 or r > 0.5 means a negative or positive correlation, respectively. Correlation significance was determined by a two‐sided Student's t‐test (*p* < 0.05). An advanced Diff VolcanoPlot plot of dominant bacterial species, which probably play crucial roles in bacterial wilt suppression in the peanut rhizosphere, was performed using the OmicStudio tools at https://www.omicstudio.cn/tool. Significantly up and down enriched bacterial species (OTUs) were identified according to their Relative Abundance (RA) between different treatments using Tukey's test (n = 3 biologically independent samples, adjusted *p* < 0.05). Based on the KEGG database, the Phylogenetic Investigation of Communities by Reconstruction of Unobserved States program was used for functional prediction of the microbiota.

### 5.4 Transcriptome Analysis

Root tissues collected from XYH108, ZZH107, and ZZH108 blocks were used for high‐throughput RNA sequencing (RNA‐seq) analysis of defense response in peanut plants against BW infection. Total RNA was extracted from peanut roots using an RNA Simple Total RNA Kit (Tiangen, Beijing, China). The integrity and quantity of the RNA were evaluated with gel electrophoresis and a Nanodrop 2000c spectrophotometer (Nanodrop, Thermo Fisher Scientific, Waltham, USA), respectively. Using the NEB Next UltraTM RNA Library Prep Kit (New England Biolabs, Beijing, China), the complementary deoxyribonucleic acid (cDNA) libraries were constructed with approximately 3 µg RNA of each sample. Subsequently, RNA‐seq was conducted with an Illumina Hi‐Seq × 10 RNA‐sequencing platform at the Metware Biotechnology Co., Ltd. (Wuhan, China).

Raw data were processed and filtered using the FastQC software (http://www.bioinformatics.babraham.ac.uk/projects/fastqc). Low‐quality RNA‐seq reads with nucleotides less than 50 and a Phred quality score of less than 20 were removed, and the remaining clean reads were then mapped onto the reference whole genome sequence of peanut (*Arachis hypogaea* Tifrunner. gnm2. ann1.4K0L) using the HISAT2 software (version 2.1.0). Quantification of the transcripts was performed with the StringTie software using the corresponding peanut genome annotation file. The expressions of genes were quantified by calculating the number and fragments per kilobase of transcript per million mapped reads (FPKM) using the StringTie software. Expression levels of genes were normalized using the Log2 (TPM+1) standardization method, and DEGs between different experimental groups were determined using the DESeq2 software (*p*< 0.05). GO and KEGG analyses were conducted using the topGO and clusterProfiler software, respectively.

WGCNA was performed using the WGCNA package in R, and genes were clustered into distinct modules based on their expression patterns. Module‐trait relationships were assessed by calculating Pearson correlations between module eigengenes and various treatments. Modules showing significant associations (|r| > 0.5, *p* < 0.05) with the BW resistance in H108 (XYH108) were considered to be biologically relevant, and DEGs in the significant module were defined as core DEGs. For visualization of the standardized results, the two software R‐4.0.2 and TB‐tools were employed [99].

### 5.5 Expression Analysis of Defense‐Related Genes by qRT‐PCR

To investigate expression profiles of key defense‐related genes, pot experiments of peanut plants with *R. solanacearum* infection or/and/or exogenous SA induction were performed. Briefly, healthy H108 seeds were soaked in sterile water at room temperature (approximately 25°C) overnight, and germinated seeds were transplanted into plastic pots (10 × 10 × 10 cm, 4 plants per pot) containing a sterilized mixture of vermiculite and nutrient substrates (3/1, v/v) in a greenhouse with suitable environmental conditions. Three‐week‐old peanut seedlings were inoculated with *R. solanacearum*, or soil‐drenching treated with an optimal concentration of SA (0.5 mm), respectively. An additional treatment of H108 seedlings simultaneously treated with *R. solanacearum* and exogenous SA was also conducted. For the *R. solanacearum* inoculation, 200‐mL bacterial pathogen suspension (10^8^ CFU/mL) was watered to each pot. Meanwhile, peanut plants were treated with an equal volume of H_2_O as a blank control. Root tissues of peanut plants, as the control, inoculated with *R. solanacearum* (R.S), treated with SA (SA), or simultaneously treated *R. solanacearum* and exogenous SA (R.S + SA), were harvested at one week post various treatments. Consequently, total RNA was extracted using an RNA Simple Total RNA Kit (Tiangen, Beijing, China), and was evaluated by agar gel electrophoresis and a Nanodrop 2000c spectrophotometer (Nanodrop, Thermo Fisher Scientific, Waltham, USA) for integrity and quantity, respectively.

Primer pairs were designed from respective cDNA sequences of specific to defense‐related genes, including *STRG.1004*, *Ah17g014300*, *Ah10g050800*, *STRG.5630*, *Ah11g187000*, *Ah19g395500*, *Ah19g396600* and *Ah11g430600* using the Primer‐BLAST software (https://www.ncbi.nlm.nih.gov/tools/primer‐blast/). The primers of those genes and the internal reference gene *AhACTIN7* (XM_025826875) were synthesized by Sangon Biotechnology Co. Ltd (Shanghai, China) (Table S6). PCR and agarose gel electrophoresis were conducted, and only primers amplifying a single product with the required length (150–250 bp) were used for consequent expression analysis.

The RNA isolated from roots of peanut plants collected from XYH108, ZZH107, and ZZH108 blocks, as well as those with *R. solanacearum* inoculation and SA treatments, were subjected to the synthesis of cDNA using the PrimeScript RT reagent Kit with a gDNA Eraser (Takara, Dalian, China). The qRT‐PCR was performed in a 20‐µL reaction volume comprising 2.0‐µL 5 × diluted cDNA (50 ng), 0.8‐µL each primer (10.0 µm), 10.0‐µL of 2 × SYBR Green I Master Mix, and 6.4 µL of sterile distilled water (Takara, Dalian, China). The following PCR cycles were used: 95°C for 5 min, followed by 40 cycles at 95°C for 15 s, 60°C for 30 s, and 72°C for 30 s, and then at 68°C for 5 min. All reactions were performed in a Bio‐Rad CFX‐96 Real‐time PCR System (Bio‐Rad, Hercules, Canada) with three technical replicates. The expression levels of those defense‐related genes were calculated using the relative quantification (2^−∆∆CT^) method [100].

### 5.6 Identification of the Ah10g050800 (PR1) in Peanut Resistance Against BW

To characterize the roles of SAR in peanut resistance against BW, phylogenetic analysis of the SAR‐related gene *PR1* (*Ah10g050800*) was conducted with the ten homologous PR1 genes from various plant species (Table S7). The predicted full‐length AA sequences of those homologous PR1 proteins were used for the construction of the neighbor‐joining phylogenetic tree using the MEGA 7.0 software, applying 1000 replicates, and the confidence values lower than 70% were cutoff.

The full‐length coding sequence of *Ah10g050800* (*PR1*) was cloned for subcellular localization analysis and functional identification against *R. solanacearum*. Briefly, gene‐specific primer pairs were designed according to the complete coding sequence of *Ah10g050800* using the Primer Premier 5.0 software (Premier, Palo Alto, CA, USA) (Table S6). The full‐length coding sequence of *Ah10g050800* without the termination codon was amplified using the H108 template cDNA and the Primer STAR Max DNA polymerase (Takara, Dalian, China). The amplified PCR fragments were gel‐purified and cloned into the binary expression vector pCambia1300‐YFP between the double 35S cauliflower mosaic virus promoter and the yellow fluorescent protein (YFP) using the CloneSmarter seamless assembly cloning kit (CloneSmarter, Houston, TX, USA). The recombinant vector pCambia1300‐Ah10g050800‐YFP was confirmed by sequencing.

The plasmids of the pCambia1300‐Ah10g050800‐YFP and the pCambia1300‐YFP (empty control) were subsequently transformed into the *Agrobacterium tumefaciens* GV3101 competent cells (containing the pSoup‐p19 vector), respectively. The *A. tumefaciens* cells harboring the pCambia1300‐Ah10g050800‐YFP and pCambia1300‐YFP, respectively, were mixed with an equal‐volume culture suspension of *A. tumefaciens* cells harboring the AtPIP2a‐RFP plasma membrane marker vector. Subsequently, the free YFP and the protein fusion Ah10g050800‐YFP were co‐expressed with the plasma membrane marker AtPIP2a‐RFP fusion by infiltrating the bacterial mixtures into fully expanded tobacco (*Nicotiana benthamiana*) leaves. After being incubated in darkness at 24°C for 24 h, the infiltrated tobacco leaves were grown in a greenhouse maintained at a temperature of 24°C with a light cycle consisting of 16 h of light (15 000 lx) followed by 8 h of darkness and humidity levels set at approximately 60%. Following an additional 24‐h culturation, fluorescence of free YFP and fusion proteins was observed under an LSM710 confocal laser microscope (Carl Zeiss, Inc., Jena, Germany). Finally, 3–5 images were randomly taken for each sample, and subcellular localization analysis was conducted according to the specific expression locations of free YFP and protein fusions.

To identify potential resistance of the *Ah10g050800* (*PR1*) against BW, wild‐type (WT) tobacco leaves and tobacco leaves overexpressing free YFP and the Ah10g050800‐YFP fusion were inoculated with *R. solanacearum*. After 3‐days’ incubation, cell death and necrotic lesions caused by *R. solanacearum* infection were viewed and indicated by TB staining. The *R. solanacearum* inoculation and TB staining were performed as previously described [16].

### 5.7 Extraction and Profiling of Rhizosphere Metabolites

The rhizosphere metabolites were extracted and determined using an untargeted liquid chromatography‐tandem mass spectrometry (LC–MS/MS). Briefly, 0.2‐g rhizosphere soil of each sample was ultrasonically homogenized in a 2‐mL Eppendorf tube containing 0.5‐mL prechilled 80% methanol and 0.1% formic acid. Following being centrifuged at 15 000 rpm for 5 min at 4°C, the supernatant was diluted to a final concentration containing 53% methanol. After being centrifuged at 15 000 *g* for 10 min at 4°C, the supernatant samples were transferred into fresh 2‐mL tubes and subjected to UHPLC‐MS/MS analysis.

The LC‐MS/MS analyses were performed in Biozeron Co., Ltd. (Shanghai, China) using a Vanquish UHPLC system (Thermo Fisher, Germany) coupled with an Orbitrap Q Exactive TM HF mass spectrometer (Thermo Fisher, Germany). Rhizosphere soil metabolites were analyzed in both positive and negative ionization modes through full MS and higher‐energy collisional dissociation data‐dependent MS/MS analysis. Using the Compound Discoverer 3.1 software (Thermo Fisher, Germany), the raw data were processed to perform peak alignment, peak picking, and quantitation for each metabolite at the Metabolomics Standards Initiative confidence level 2. Metabolites were annotated using the massbank (http://www.massbank.jp/), LipidMaps (http://www.lipidmaps.org), Human Metabolome Database (http://www.hmdb.ca/metabolites), and self‐built standard product databases. The in‐house database contains retention time, MS/MS spectra, and other information for authentic standards, covering major classes of primary and secondary metabolites. PCA and Partial least squares discriminant analysis were performed using metaX, a comprehensive software for processing metabolomics data. The DEMs were determined using a combination of a statistically significant threshold for variable influence on projection (VIP) values obtained from a PLS‐DA model and *p*‐values from a two‐tailed Student's t‐test on the normalized peak areas from different samples. VIP values greater than 1.0 and fold change (FC) ≥ 2 or FC ≤ 0.5 denoted DEMs, and a *p*‐value < 0.05 was considered statistically significant.

Volcano plots were used to filter crucial metabolites based on their log 2 (FC) and ‐log10 (*p*‐value). WGCNA of metabolites was performed using the WGCNA package in R, and genes were clustered into distinct modules based on their relative abundance. Modules showing significant associations (r > 0.5, *p* < 0.05) with the XYH108 were considered to be biologically relevant, and DAMs in the significant module were defined as core DAMs. The metabonomic data were explored for the accumulation level analysis of DEMs, and the TBtools were utilized for data visualization.

Pearson's correlation analysis was performed using the OmicStudio Advanced Cor link tools (https://www.omicstudio.cn/tool) to investigate the association relationships between the relative abundance of DAMs and beneficial bacterium species, as well as between the relative abundance of DAMs and relative expression levels of defense‐related genes. Correlation pairs of *p* < 0.05 and absolute r ≥ 0.3 are considered to be statistically significant correlated, and r > 0 means positive correlation, while r < 0 means negative correlation.

### 5.8 Investigation of the Effects of Commercial Metabolites to Peanut BW

To investigate the effects of commercial metabolites on BW‐infected peanut plants, hydroponic and soil‐pot experiments were simultaneously carried out for root and soil‐drenching applications of metabolites, respectively. For the hydroponic experiments, germinated seeds of the BW‐susceptible genotype H107 were grown in the 1/2 Hoagland's solution in hydroponic pots (20 × 10 × 20 cm, 12 plants per pot) in a greenhouse with suitable environmental conditions. Three weeks later, the hydroponic peanut plants were inoculated with *R. solanacearum* using a root‐tip cutting method [15], followed by root application of 10‐mM BTA, 2‐mg/mL Arg, 0.5‐mM SA, and 15‐µg/mL TC, respectively. Root application of those metabolites was performed by adding the corresponding metabolites into the 1/2 Hoagland's solution. For the soil‐pot (Soil‐drenching application) experiment, germinated H107 seeds were planted in healthy soils in pots (10 × 10 × 10 cm, 4 plants per pot) in a greenhouse with suitable environmental conditions.

Three‐week‐old H107 seedlings were inoculated with *R. solanacearum* by watering 200‐mL bacterial pathogen suspension (10^8^ CFU/mL) to each pot. Meanwhile, soil‐drenching application of those metabolites was conducted by watering water solutions containing 10‐mM BTA, 2‐mg/mL Arg, 0.5‐mm SA, and 15‐µg/mL TC, respectively. Application of equal volume H_2_O (100 mL per plant) was conducted as a blank control. For each treatment, three and nine pots (3 replications) were carried out for root and soil‐drenching applications. The growth and disease incidence of BW‐diseased peanut plants in each treatment were investigated at 14 dpi [16]. Bacteriostasis rate of those metabolites against *R. solanacearum* was tested by counting the colonies on the TTC agar medium containing 10‐mM BTA, 2‐mg/mL Arg, 0.5‐mM SA, and 15‐µg/mL TC, respectively. The TTC agar medium without metabolites was considered the blank control.

### 5.9 Development and Laboratory Test of the Prebiotics

The prebiotic formulation was developed by equally mixing the solutions containing 10‐mm BTA, 2‐mg/mL Arg, and 0.5‐mM SA concerning about the ecological safety, practicability, and cost. An in vitro bacteriostasis test was conducted to evaluate the inhibiting of the prebiotics to the pathogen *R. solanacearum*. Additionally, root and soil‐drenching applications of the prebiotics were also conducted to investigate the effects of the prebiotics on BW‐infected peanut plants. Hydroponic and soil‐pot experiments were simultaneously carried out, and the disease incidence of BW‐diseased peanut plants in each treatment was investigated at 14 dpi following the above methods. Additionally, the root bacterium amounts of *R. solanacearum* were determined as Zhao et al. described [24]. Application of H_2_O was conducted as a blank control, and three pots (3 replications, 12 plants per pot) were carried out for each treatment.

Furthermore, the prebiotics were also tested for promoting peanut resistance against the fungal stem rot pathogen *S. rolfsii*. Germinated seeds of H107 were planted into culture medium with vermiculite and nutrient substrates (3/1, v/v) in plastic pots (10 × 10 × 10 cm, 24 plants per pot) in a greenhouse with favorable environmental conditions. The autoclaved oats (*Avena sativa* L.) seeds were inoculated with the *S. rolfsii* mycelium for 3‐day incubation at 28°C, and equal oats seeds covered with mycelium were placed near the stem of peanut plants [16]. Meanwhile, the peanut plants inoculated with the pathogen *S. rolfsii* and mock control (equal autoclaved oats without mycelium) were daily sprayed with the prebiotics, and the disease incidence of stem rot was investigated at 1, 2, 3, 4, and 5 dpi. Three replications were carried out for each treatment. Moreover, the inhibition of the prebiotics to the growth of the fungal pathogen was also investigated by measuring the colony diameter of the *S. rolfsii on* the potato dextrose agar medium containing the prebiotics and H_2_O (blank control).

### 5.10 Field Test of the Prebiotics to the Peanut Plants Under BW Infection

To test the practical capacity of the prebiotics, field experiments were performed in the BW‐prevalent field at Xinyang and the healthy field at Zhengzhou. To investigate the effects of the prebiotics on promoting peanut resistance against BW, H107 and H108 were planted in the BW‐diseased soil, and three‐week‐old seedlings were inoculated with *R. solanacearum* as mentioned above. Meanwhile, the two genotypes were also planted in the *R. solanacearum*‐free soil as mock controls.

Before being sown in the fields, the seeds of H107 and H108 were soaked in the prebiotics and H_2_O (blank control) overnight. In addition, twice soil‐drenching applications of the prebiotics were conducted at 15‐ and 30‐day post seedling emergence. Collectively, four treatments, including the blank control, the prebiotics‐treated (Prebiotics), the *R. solanacearum*‐infected (R.S), and the *R. solanacearum*‐infected with prebiotics‐treated (R.S + Prebiotics) were conducted for H107 and H108, respectively. All treatments were conducted in a randomized block design with three biological replications. Those blocks were arranged and managed as mentioned above.

The final survival rates of H107 and H108 plants infected with *R. solanacearum* were investigated at 80 days post sowing. Additionally, the effects of the prebiotics on the growth and yield of peanuts were investigated by measuring relevant agronomic traits, including the main stem length, main branch length, branch number, needle number, pod number, full‐pod number per plant, pod weight per plant, hundred pod weight, hundred seed weight, and pod yield.

To explore the underlying mechanisms of the prebiotics in protecting peanuts from BW, the 16S rRNA microbial diversity analysis of the H107 rhizosphere soil was performed again as mentioned above. In addition, the roots of H107 plants with the control, prebiotics, R.S, and R.S + prebiotics treatments were collected at 7 days post the first prebiotics soil‐drenching application treatment, and the regulation of the prebiotics to the defense‐related genes was investigated by qRT‐PCR.

### 5.11 Statistical Analysis

All experiments were conducted with at least three independent biological replicates. The statistical analysis was performed using SPSS software package (version 18.0), and the data are average values of at least three (or five) biological replicates, and error bars indicate standard deviation. ^*^ and ^**^ represent *p* <0.05 and *p* <0.01, and the significant differences were assessed by two‐sided Student's *t*‐test; different letters (a–h) indicate significant differences at *p* <0.05 based on the Tukey–Kramer test.

## Author Contributions

R.R., and X.G.L. contributed equally to this work. D.M.Y., X.L.M., and X.X.W. conceived and designed this research; R.R., H.L., Z.H.C., M.T.H., C.L.Z., N.L., S.S.H., Y.Z.L., Q.M., Y.Y.L., Y.F., K.K.Z., and K.Z. performed experiments; R.R., and X.G.L. wrote the paper; D.M.Y., X.X.W., H.T.L. D.Q., F.P.G., and Z.F.L. oversaw the entire study. All authors read and approved the manuscript.

## Funding

Key Program of National Natural Science Foundation of China (NSFC)‐Henan United Fund (No. U22A20475), Zhongyuan Scholars in Henan Province (264000510007), the Key Scientific and Technological Project of Henan Province (No. 221111110500; HARS‐22‐05‐G1; No. 262102111109; 242102111154), and the Special Fund for Young Talents in Henan Agricultural University (30501308/111).

## Conflicts of Interest

The authors declare no conflicts of interest.

## Supporting information




**Supporting File 1**: advs75910‐sup‐0001‐SuppMat.docx.

**Supporting File 2**: advs75910‐sup‐0002‐table.zip.

## Data Availability

The data that support the findings of this study are available from the corresponding author upon reasonable request.
