# Supplementary material for: Resistant Peanut Genotype Reprograms Rhizosphere Metabolism to Enhance Bacterial Wilt Suppression
Source: Adv Sci (Weinh). 2026 Jun 1;13(48):e75910. doi: 10.1002/advs.75910 (PMC13336948; doi:10.1002/advs.75910)
Supplement: Supplementary file 1 [file ADVS-13-e75910-s001.docx]

**Figure S1.** 16S rRNA bacterial diversity analysis of the XYH107, XYH108, ZZH107 and ZZH108. (A) PcoA analysis of the rhizosphere bacterial communities of the four treatments. (B) Bacterial community diversity indicated by the Chao1, the ACE and the Simpson indexes. Boxplots indicate median (middle line), percentiles (box), and maximum and minimum values (whiskers) (n = 3 individual replicates), and each data point represents a biologically independent replicate. Different letters (a-c) indicate significant differences among treatments (Tukey’s test, *p* < 0.05). (C) RA (%) of the top 10 dominant phyla in the rhizosphere bacterial communities of the four groups. (D) Bubble chart of the top 45 bacterial species with significant different RA (%) identified in rhizosphere bacterial communities of the four groups; Red characters indicate the pathogen *R. solanacearum*, and the cyan characters represent reported beneficial bacterial species. The size and color of the dots in the figure correspond to the average RA (%) of the species in the corresponding group; The lowercase letters in the circles represent the results of the significance test for the differences in species abundance between groups (Tukey’s test, *p* < 0.05).

**Figure S2.** Cross‑phyla co-occurrence network of the core OTUs. In total of 77 OTUs were selected out of the 476 core OTUs for construction of a cross‑phyla co-occurrence network. The 77 core OTUs were selected according to the fold change (FC) of their relative abundance (RA) in XYH107 vs. XYH108 (|FC| > 4.0). The network was constructed according to the Spearman’s correlation coefficients (r), and r < -0.5 or r > 0.5 mean negative (cyan lines) or positive (red lines) correlation, respectively. Solid lines indicate significant correlation determined by two-sided Student’s t-test (*p* < 0.05).

**Figure S3.** Predicted functions of rhizosphere bacterial communities of the XYH107, XYH108, ZZH107 and ZZH108.

**Figure S4.** GO analysis of DEGs between the XYH108 vs. XYH107, XYH108 vs. ZZH108, ZZH108 vs. ZZH107 and XYH107 vs. ZZH107 compare groups.

**Figure S5.** KEGG analysis of the DEGs identified in the XYH108 vs XYH107, XYH108 vs ZZH108, ZZH108 vs ZZH107 and XYH107 vs ZZH107 compare groups.

**Figure S6.** Expression profiles of pathogenesis-related proteins and DEGs related plant defense response. Colors of the mosaics indicate gene expression level values ranging from 0-18.0 (average value of 3 individual replicates).

**Figure S7.** Expression analysis of key genes related to GSH metabolism and plant-pathogen interaction in response to *R. solanacearum* infection and SA induction. Data are shown as means±SD, and different letters (a-f) indicate significant differences among treatments (n=3 biologically independent samples, Tukey’s test, *p* < 0.05).

**Figure S8.** KEGG analysis of the DAMs identified in the four compare groups.

**Figure S9.** Association analysis of the 12 key DAMs with the pathogen *R. solanacearum*. Correlation pairs of *p* < 0.05 and absolute r ≥ 0.3 is considered to be statistically significant correlated, and r > 0 means positive correlation (red lines) while r < 0 means negative correlation (cyan lines).

**Figure S10.** Effects of the prebiotics on the suppression of *R. solanacearum* and *S. rolfsii*. (A) Effects of the prebiotics on the suppression of *R. solanacearum* in the hydroponics experiment. (B) Effects of the prebiotics application on the root bacterium of hydroponics and soil-culture H107 plants under *R. solanacearum* infection. Each data point represents a biologically independent replicate, and data are shown as mean ± SD (n = 3). Variations with significant difference among treatments were determined by Student’s *t*-test, and single asterisk (*) represents *p* < 0.05 and double asterisks (**) indicate *p* < 0.01. (C) Effects of the foliar application of the prebiotics to the disease indexes of H107 to the fungal stem rot disease caused by the *S. rolfsii*. d *In vitro* antibacterial tests of the prebiotics on the diameter of *S. rolfsii* colonies.

**Figure S11.** Effects of the prebiotics application on the agronomic traits of peanuts in the diseased and healthy filed. Each data point represents a biologically independent replicate, and data are shown as mean ± SD (n = 5). Variations with significant difference among treatments were determined by Student’s *t*-test and indicated with * (*p* < 0.05) and ** (*p* < 0.01). ns indicates no significant difference.

**Figure S12.** Diversity analysis of the rhizosphere bacterial communities of H107 in response to the application of prebiotics under BW infection. (A) PcoA analysis of the rhizosphere bacterial communities. (B) Diversity of rhizosphere bacterial communities indicated by the Chao1, the ACE and the Simpson indexes. Boxplots indicate median (middle line), percentiles (box), and maximum and minimum values (whiskers) (n = 3 individual replicates), and each data point represents a biologically independent replicate. Different letters (a-c) indicate significant differences among treatments (Tukey’s test, *p* < 0.05). (C) The 35170 OTUs identified in the four groups. (D) Functions prediction of the rhizosphere bacterial communities. (E) Bacterial species with significantly differences between the prebiotics and CK group. The cyan characters represent reported beneficial bacterial species. Each data point represents average Log_2_FC of bacterial species with significant differences between different treatments (3 biologically independent samples, Tukey’s HSD test, adjusted *p* < 0.05).

**Figure S13.** RA of rhizosphere bacterium enriched across the control, Prebiotics, R.S and R.S + Prebiotics samples. Red labels denote *R. solanacearum*, and cyan labels denote reported beneficial taxa.
